# Tumor-Informed ddPCR for Personalized Longitudinal ctDNA Monitoring in Advanced Solid Tumors: Molecular–Radiological Concordance and Molecular Lead Time

**DOI:** 10.3390/ijms27188340

**Published:** 2026-09-19

**Authors:** Dadasaheb Akolkar, Sneha Puranik, Sachin Apurwa, Shivani Chaudhari, Pratiksha Nandre, Swapnil Puranik, Vinayak Rao, Raja Dhasarathan, Navin Srivastava, Darshana Patil, Vineet Datta, Prashant Kumar, Raymond Page, Stefan Schuster, Ajay Srinivasan, Rajan Datar

**Affiliations:** 1 Research and Innovations, Datar Cancer Genetics (Pvt) Ltd., Nasik 422010, MH, India; sneha.puranik@datarpgx.org (S.P.); sachinapurwa@datarpgx.org (S.A.); chaudhari.shivani33@gmail.com (S.C.); pratikshanandre04@gmail.com (P.N.); labadmin@datarpgx.org (S.P.); bioinformatics@datarpgx.org (V.R.); navinshrivastava@datarpgx.org (N.S.); drdarshanap@datarpgx.com (D.P.); drvineetdatta@datarpgx.com (V.D.); prashant.agrawal@datarpgx.com (P.K.); ajays@datarpgx.org (A.S.); rajandatar@datarpgx.com (R.D.); 2 Quality Assurance, Datar Cancer Genetics UK (Pvt) Ltd., Guildford GU2 7YD, UK; draja@datarpgx.org; 3 Biomedical Engineering, Worcester Polytechnic Institute, Worcester, MA 01609, USA; rpage@wpi.edu; 4 Research and Innovations, Datar Cancer Genetics Europe GmbH, 95447 Bayreuth, Germany; drstefanschuster@datarpgx.com

**Keywords:** circulating tumor DNA, droplet digital PCR, tumor burden, RECIST, treatment response, liquid biopsy, molecular residual disease, molecular lead time, variant allele fraction

## Abstract

Serial circulating tumor DNA (ctDNA) monitoring may complement episodic radiological response assessment during systemic therapy. We evaluated personalized tumor-informed droplet digital PCR (ddPCR) for longitudinal ctDNA monitoring in advanced solid tumors, focusing on RECIST-associated ctDNA levels, anatomical tumor burden, molecular–radiological concordance, molecular lead time (MLT), and routine implementation. The clinical cohort comprised 61 patients with breast, colorectal, gynecological, head and neck, lung, pancreaticobiliary, or other advanced solid tumors and 385 longitudinal patient-specific ddPCR assessments; 357 assessments were imaging-concurrent. Repeated RECIST-stratified measurements were evaluated descriptively and using patient-clustered generalized estimating equations (GEEs). Longitudinal trajectories were classified using study-specific Longitudinal Concordance Level (LCL) and Molecular Evidence Level (MEL) frameworks. A separate real-world implementation cohort comprised 270 patients and 650 personalized ddPCR assays; 88 patients underwent two or more serial assessments. Among the 357 imaging-concurrent assessments, 90 were partial response (PR), 117 stable disease (SD), and 150 progressive disease (PD). Median VAF was 0.055% in PR, 0.190% in SD, and 0.205% in PD. Patient-clustered GEEs confirmed an overall association between VAF and RECIST category (Wald χ^2^ = 12.60, *p* = 0.00183), with lower VAF in PR than SD (Holm-adjusted *p* = 0.00119) or PD (*p* = 0.00247), but no separation between SD and PD (*p* = 0.539). In 210 imaging–ctDNA pairs from 53 patients, correlations with estimated tumor volume (ρ = 0.169) and RECIST SLD (ρ = 0.177) were weak. Of 50 evaluable longitudinal trajectories, 21 (42.0%) were LCL-1. Among 14 patients who developed radiological PD after an initial PR or SD, molecular progression preceded radiological progression in 9 (64.3%), was concurrent in 3 (21.4%), and no qualifying pre-radiological molecular-progression event was identified in 2 (14.3%); positive MLTs had a median of 55 days (IQR 46–83; range 28–209). The real-world cohort included five genomic alteration classes and a median assay-specific LoD of 0.020% VAF (IQR 0.020–0.030%). Personalized tumor-informed ddPCR showed population-level association with RECIST response and structured longitudinal molecular–radiological relationships, while substantial VAF overlap and weak anatomical-burden correlations limit interpretation of isolated measurements. Molecular progression preceded imaging-defined progression in a subset of patients, supporting prospective evaluation of ctDNA-triggered reassessment rather than establishing benefit from ctDNA-guided intervention. The separate real-world cohort supports operational feasibility across diverse assay designs.

## 1. Introduction

Accurate, real-time assessment of tumor response during systemic therapy remains a fundamental challenge in clinical oncology. Radiological imaging is the established standard for treatment response assessment but is episodic, primarily reflects anatomical rather than biological changes, and may lag treatment response. It is also limited by radiation exposure, reduced sensitivity for micro-metastases [1,2], and interference from inflammation, postsurgical changes, or necrosis [3]. In clinical trials, tumor burden is commonly assessed using Response Evaluation Criteria in Solid Tumors (RECIST v1.1) [4,5], which relies on unidimensional measurements of selected target lesions and is susceptible to inter-observer variability and lesion-selection bias. Furthermore, conventional RECIST may inadequately characterize responses to immunotherapy because atypical patterns, including pseudo-progression and delayed response, can create discordance between early radiological findings and subsequent clinical or biological disease behavior [6]. Importantly, imaging provides no direct information on the molecular evolution of cancer.

Circulating tumor DNA (ctDNA), comprising tumor-derived cell-free DNA fragments carrying somatic genomic alterations, provides a complementary molecular biomarker of tumor dynamics [7,8]. ctDNA shedding correlates with tumor stage, metastatic burden, anatomical site, and proliferative activity [9,10]. Tumor-informed or tumor-specific assays detect ctDNA in approximately 80–85% of pretreatment patients with advanced or mixed-stage solid tumors, although detection varies by tumor type, stage, disease burden, and treatment status; detected VAFs may span several orders of magnitude [11,12].

Clinical applications of ctDNA are being actively developed for early cancer detection, molecular residual disease assessment, and surveillance for molecular recurrence [13]. In contrast, longitudinal ctDNA monitoring during active systemic therapy remains comparatively underexplored [14], despite evidence that molecular progression may precede radiological progression by weeks to months [15,16].

Recent research has also emphasized the need for standardized interpretation of on-treatment ctDNA kinetics. The RECIST Working Group has identified plasma ctDNA response as a promising treatment-response biomarker while highlighting unresolved questions concerning sampling time, assay thresholds, cancer-specific effects, and prospective validation [17]. A complementary proposal, ctDNA-RECIST, classifies serial ctDNA changes relative to measurement uncertainty and is now undergoing prospective clinical evaluation [18].

Serial ctDNA monitoring requires reproducible quantification of low-abundance tumor-derived variants across repeated time points. Tumor-informed strategies identify patient-specific somatic alterations from tumor or baseline molecular profiling and can then use highly targeted and sensitive [19] digital PCR assays for longitudinal measurement of predefined or patient specific variants [20]. This approach supports analytical specificity by minimizing confounding from passenger variants, clonal hematopoiesis of indeterminate potential (CHIP), and germline polymorphisms. This focused strategy is well suited to frequent quantitative monitoring of selected molecular signals but, because a single variant is followed, does not capture the full spectrum of clonal evolution or newly emergent resistance alterations. Prospective multi-histology studies integrating personalized ddPCR with serial radiological assessment and molecular lead-time analysis remain limited. We have previously demonstrated the feasibility of longitudinal ddPCR monitoring in non-small cell lung cancer [21] and periampullary carcinoma [22].

Liquid biopsy also encompasses circulating tumor cells (CTCs), which retain intact cellular material and can provide phenotypic, genomic, and potentially functional information that is complementary to the acellular genomic signal provided by ctDNA [23,24]. The present study specifically evaluates ctDNA and does not compare liquid-biopsy analytes.

Here, we report a biomarker sub-study of a clinical trial [25] evaluating personalized tumor-informed ddPCR in patients with advanced solid tumors receiving systemic therapy. We assessed concordance between serial ctDNA VAF and RECIST response categories, quantified associations with radiological tumor burden using estimated tumor volume and RECIST sum of longest diameters (SLD) and determined whether molecular progression preceded radiological progression [26] using a pre-specified longitudinal algorithm. A separate real-world implementation cohort was analyzed to evaluate operational feasibility, assay scalability, and ctDNA detection across diverse tumor types and genomic alteration classes, providing implementation context for future prospective ctDNA-guided studies.

## 2. Results

### 2.1. Study Cohort Characteristics and Assay Performance

Sixty-two patients were screened for inclusion in the (clinical) longitudinal ctDNA biomarker cohort. Of these, 61 were participants in the RESILIENT clinical trial (CTRI/2018/02/011808) and one was derived from an observational cohort. One clinical-trial participant was excluded at the outset because the candidate (patient-specific) tracking variant was confirmed as CHIP-derived by matched leukocyte testing (see CHIP Exclusion Protocol). The remaining 61 patients constituted the clinical analytical cohort. Plasma specimens from the clinical trial were collected between Dec 2017 and May 2019, while those from the observational-cohort patient were collected between Nov-2016 and Dec 2017. For one trial participant, additional pre-trial plasma specimens were available between Nov 2017 and Dec 2017. For reporting convenience, the observational-cohort patient was grouped with the trial patients. Baseline demographic and clinical characteristics (n = 61) are summarized in Table 1.

A total of 385 longitudinal ctDNA assessments were performed across the cohort, corresponding to a median of 6 assessments per patient (IQR 4–8; range 2–19). The median interval between consecutive ctDNA assessments was 32 days (IQR 26–43 days). All assessments met predefined quality control criteria, yielding an assay success rate of 100%. Monitoring was performed using 36 unique personalized ddPCR assay designs targeting 13 cancer-associated genes, including TP53 (n = 21, 34.4%), PIK3CA (n = 14, 23.0%), KRAS (n = 7, 11.5%), GNAS (n = 5, 8.2%), EGFR (n = 4, 6.6%), FGFR3 (n = 2, 3.3%), AKT1 (n = 1, 1.6%), APC (n = 1, 1.6%), BRCA2 (n = 1, 1.6%), CDKN2A (n = 1, 1.6%), IDH1 (n = 1, 1.6%), NOTCH1 (n = 1, 1.6%), and NRAS (n = 1, 1.6%).

**Figure 1:**
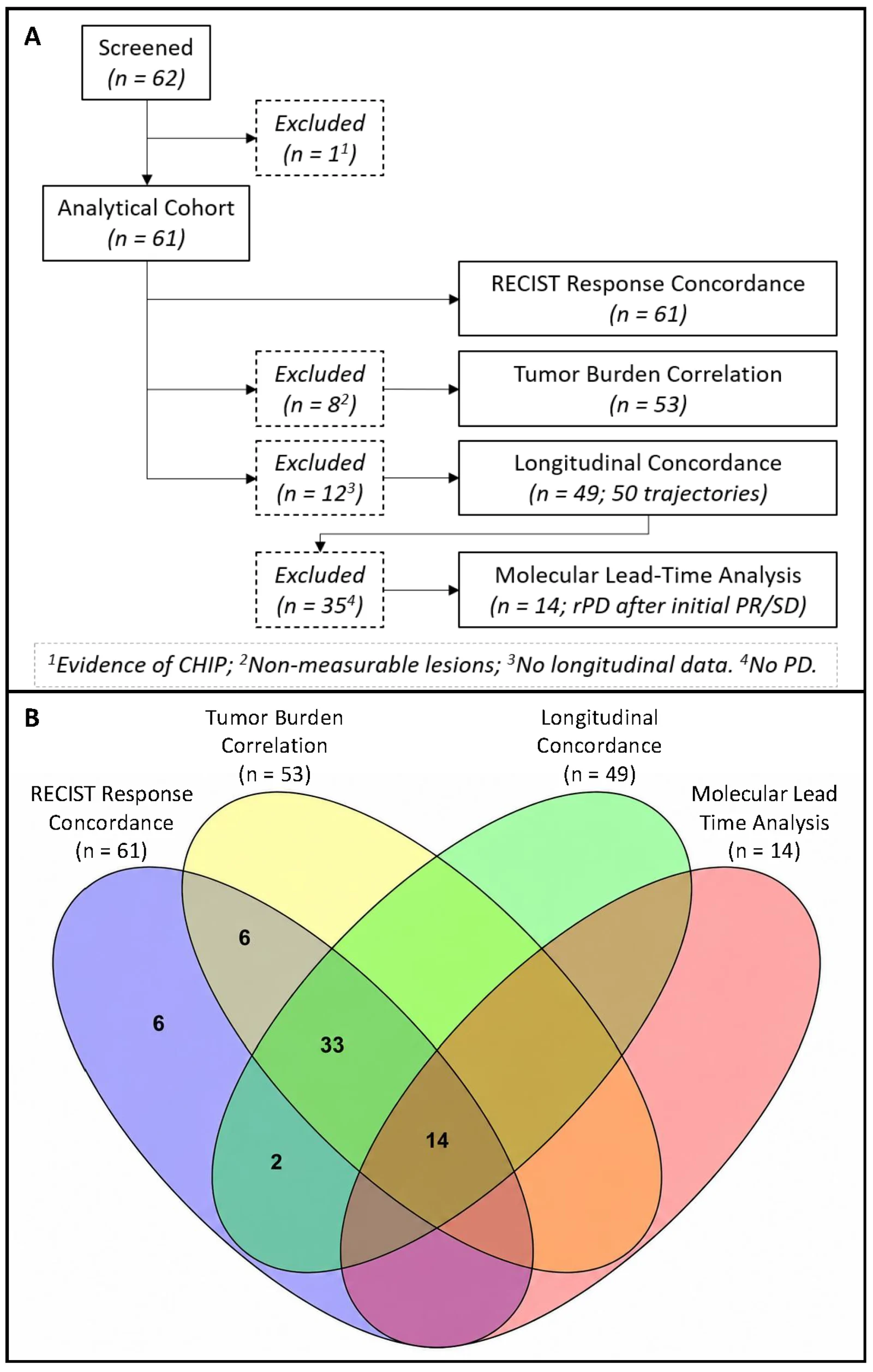
Analysis sub-cohorts reported in the study. (**A**) Study Flow Diagram. Of 62 patients initially screened, one was excluded at screening because the selected tracking variant was attributed to clonal hematopoiesis of indeterminate potential (CHIP). All remaining 61 patients in the clinical analytical cohort contributed to the RECIST response-concordance analysis. Of them, 53 had evaluable paired radiological tumor-burden measurements, and 49 patients contributed 50 evaluable longitudinal trajectories, with one patient (PID 13770) contributing two distinct treatment episodes. Fourteen patients who subsequently developed RECIST-defined progressive disease (rPD) after an initial partial response (PR) or stable disease (SD) comprised the molecular lead-time analysis cohort. (**B**) Venn Diagram. Graphical representation of the overlapping and nested analysis sub-cohorts.

**Figure 2:**
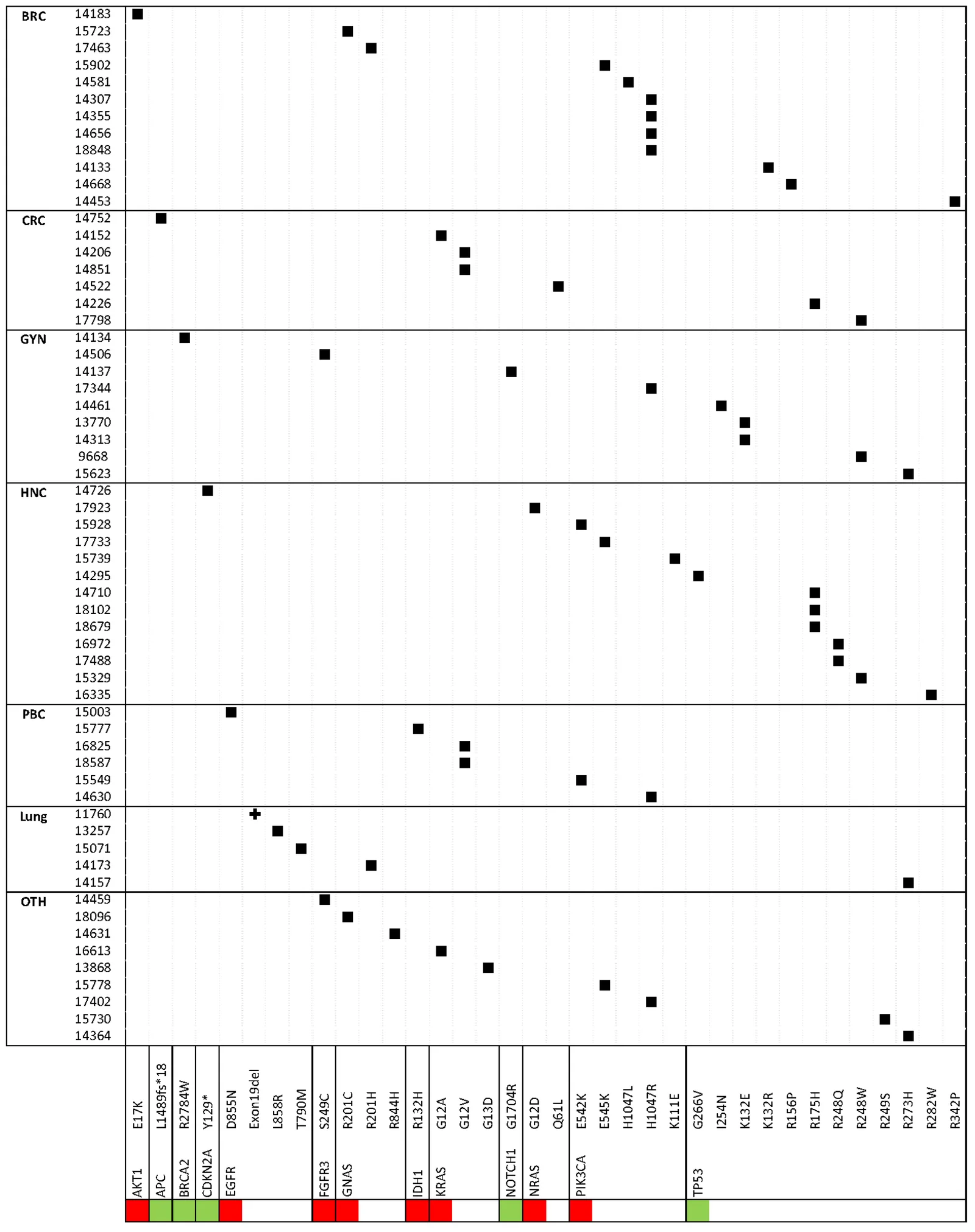
Patient-specific ddPCR assay targets in the clinical cohort. Oncoplot showing the 36 unique personalized ddPCR assay targets used for longitudinal ctDNA monitoring in the 61-patient clinical cohort. Rows represent individual patients denoted by unique 5-digit patient identifiers (PID), ordered by tumor group [breast cancer (BRC), colorectal cancer (CRC), gynecological cancers (GYN), head and neck cancer (HNC), pancreaticobiliary cancers (PBC), lung cancer, and other malignancies (OTH)]. Columns represent individual gene-variant assay targets and are grouped by gene, with separators distinguishing gene groups. The asterisk (*) is the standard symbol to denote a premature stop codon (a nonsense mutation). A black square (■) denotes a single-nucleotide variant (SNV) assay, and a plus sign (**+**) denotes the single insertion/deletion (indel) assay. Colored squares under the gene names indicate the principal functional class: red, oncogene; green, tumor-suppressor gene. Each patient was monitored using one patient-specific ddPCR assay.

Figure 1 provides a study-flow diagram (1A) and a Venn Diagram (1B) for the clinical study participants whose data were analyzed in this study. The distribution of patient-specific tracked genes and alteration classes across the 61-patient clinical cohort is summarized in Figure 2. The median assay-specific limit of detection was 0.030% VAF (range 0.010–0.540%). Across the entire cohort, baseline ctDNA VAF demonstrated substantial inter-patient heterogeneity, with a median VAF of 0.52% (IQR 0.05–3.41%; range 0–59.8%). Among baseline ctDNA-positive patients, the median VAF was 0.86% (IQR 0.21–4.72%; range 0.01–59.8%).

### 2.2. Baseline ctDNA Positivity and Serial VAF Dynamics

**Figure 3:**
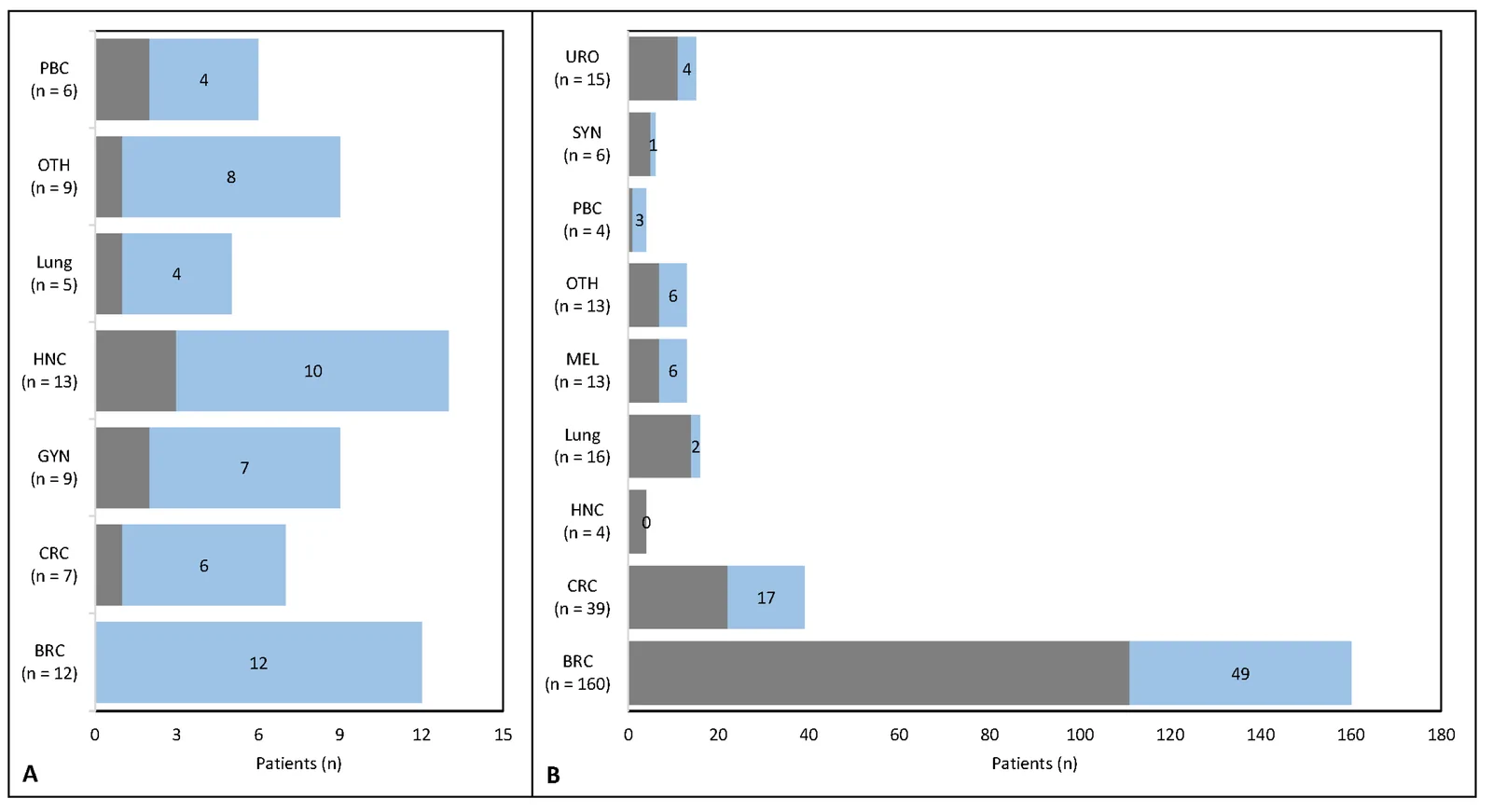
Baseline ctDNA positivity by tumor group in the clinical (**A**) and real-world (**B**) cohorts. Stacked horizontal bar plots showing patient-level baseline ctDNA status stratified by tumor group in the clinical cohort (CC; (**A**)) and the separate real-world cohort (RWC; (**B**)). The total number of patients in each tumor group is indicated within parentheses beside the corresponding category. Blue segments represent patients with detectable ctDNA at baseline (‘baseline positive’) and grey segments represent patients without detectable ctDNA (‘baseline negative’); absolute counts are displayed within the ‘baseline positive’ segments. In the CC, baseline status was determined from the patient-specific assay used for longitudinal monitoring. In the RWC, Time Point 1 was defined as baseline; because individual patients could have multiple personalized assays at baseline, a patient was classified as baseline positive when at least one Time Point 1 assay yielded a positive adjudicated ddPCR result. Each patient contributed once to the patient-level baseline classification. Tumor-group abbreviations: BRC, breast cancer; CRC, colorectal cancer; GYN, gynecological cancers; HNC, head and neck cancer; Lung, lung cancer; MEL, melanoma; OTH, other malignancies; PBC, pancreaticobiliary cancers; SYN, sarcoma; URO, urological cancers.

At baseline, ctDNA was detectable in 51 of 61 evaluable patients (83.6%), while 10 patients (16.4%) had no detectable variant. Baseline ctDNA status by tumor group is shown descriptively in Figure 3; ctDNA positivity by tracked variant is provided in Supplementary Table S1.

Because several strata contained few patients, these differences should not be interpreted as evidence that baseline detectability was causally determined by tumor type or the tracked variant. Serial VAF measurements demonstrated substantial quantitative changes throughout longitudinal follow-up and were generally concordant with clinical disease trajectory. Declining ctDNA levels were frequently observed in patients deriving clinical benefit from therapy, whereas increasing or recurrent ctDNA signals were commonly associated with disease persistence or progression. Longitudinal ctDNA monitoring captured a broad spectrum of molecular response patterns, ranging from durable ctDNA suppression to gradual molecular relapse and rapid molecular progression.

### 2.3. ctDNA VAF Stratification Across RECIST Response Categories

Of the 385 ctDNA assessments from 61 patients, 357 were imaging concurrent while 28 did not have a matched radiological assessment. To evaluate the relationship between ctDNA burden and radiographic tumor response, these 357 imaging-concurrent ctDNA assessments were stratified according to RECIST v1.1 response category, comprising 90 partial response (PR), 117 stable disease (SD), and 150 progressive disease (PD) observations. A non-zero VAF was measured in 78/90 PR (86.7%), 102/117 SD (87.2%), and 128/150 PD (85.3%) assessments, indicating comparable frequencies of non-zero measured signals across response categories. These descriptive frequencies are distinct from formally positive LoD-based assay calls. Thus, differences between groups primarily reflect quantitative variation in ctDNA burden rather than differences in variant detectability. ctDNA VAF differed significantly across response groups (Kruskal–Wallis H = 19.09, *p* = 0.000072). Median VAF increased progressively with worsening radiographic response, from 0.055% (IQR 0.025–0.218%) in PR to 0.190% (IQR 0.040–1.690%) in SD and 0.205% (IQR 0.040–6.775%) in PD. The increasing interquartile range across response categories also indicated greater heterogeneity in ctDNA burden among patients with less favorable radiographic outcomes. Post hoc Dunn’s tests with Holm correction demonstrated significantly lower VAFs in PR compared with both SD (adjusted *p* = 0.00365) and PD (adjusted *p* = 0.000048), whereas SD and PD did not differ significantly (adjusted *p* = 0.2608). Corresponding Cliff’s delta effect sizes indicated a small separation between PR and SD (δ = −0.264), a negligible separation between SD and PD (δ = −0.087), and a moderate separation between PR and PD (δ = −0.324). The above findings are summarized in Table 2.

Because individual patients contributed repeated imaging-concurrent assessments, the RECIST-stratified analysis was complemented by a patient-clustered GEE model using log10(VAF% + 0.001). The overall association between RECIST category and VAF remained significant after accounting for within-patient correlation (Wald χ^2^ = 12.60, df = 2, *p* = 0.00183). Relative to PR, VAF was higher in SD (β = 0.706 log10 units, 95% CI: 0.315, 1.096; Holm-adjusted *p* = 0.00119) and PD (β = 0.776, 95% CI: 0.305, 1.247; Holm-adjusted *p* = 0.00247), whereas PD and SD did not differ (β = 0.070, 95% CI: −0.154, 0.295; Holm-adjusted *p* = 0.539). Thus, the patient-clustered analysis confirmed population-level separation of PR from SD and PD but did not support discrimination between SD and PD.

As a complementary absolute quantification analysis, mutant copies per mL of plasma were available for all 357 imaging-concurrent assessments and showed the same overall population-level pattern as VAF (Supplementary Table S2). VAF was retained as the primary molecular metric because the prespecified longitudinal algorithms, assay-specific LoD framework, and locked analyses were defined in VAF; absolute mutant copies/mL were evaluated as a sensitivity descriptor of tumor-derived molecular abundance.

**Figure 4:**
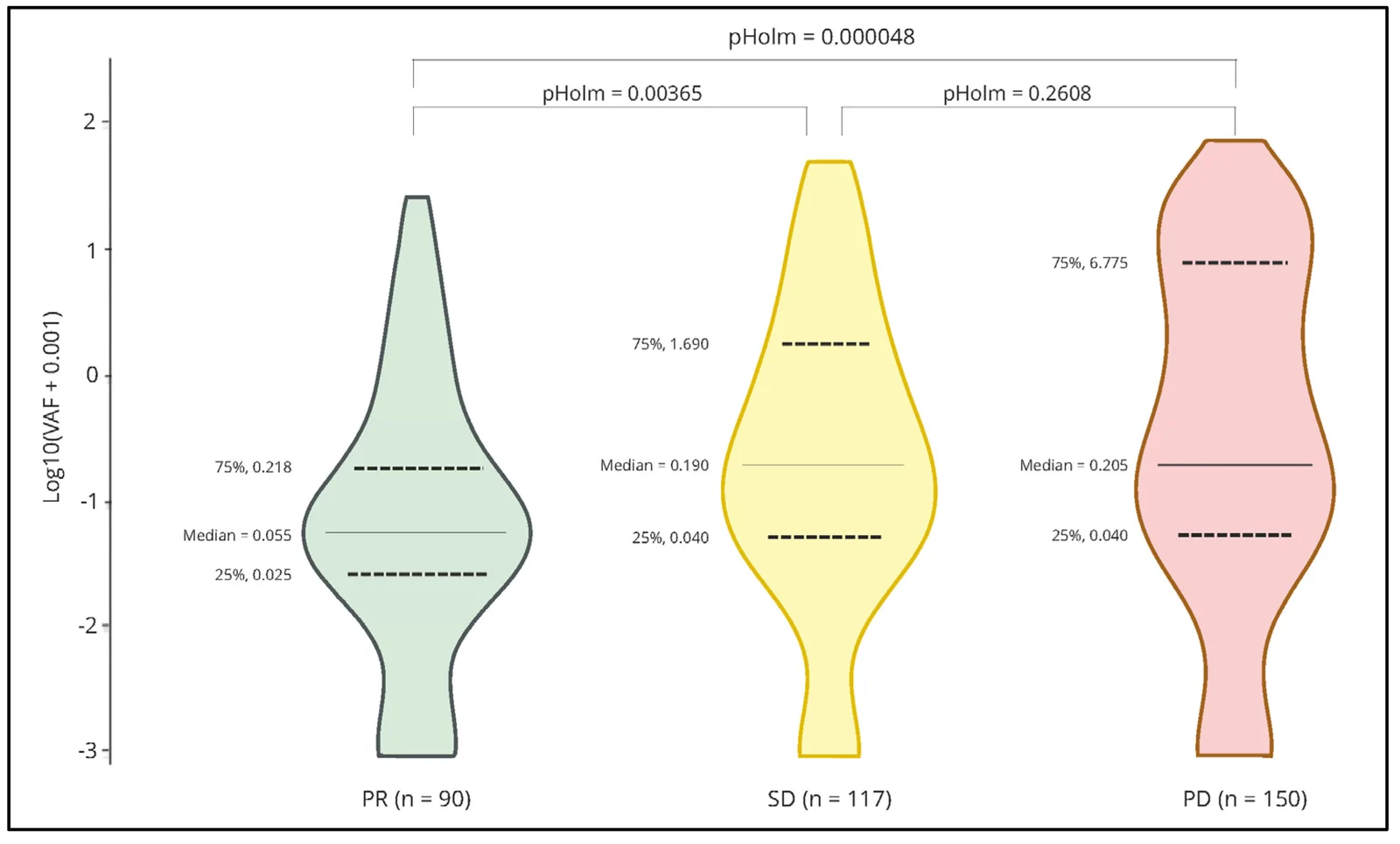
ctDNA VAF stratified by RECIST response category. Violin plots showing the distribution of ctDNA variant allele fraction (VAF; log_10_[VAF% + 0.001], Y-axis) across imaging-concurrent time points classified as partial response (PR, n = 90), stable disease (SD, n = 117), or progressive disease (PD, n = 150). The Y-axis range spans (minus) 3 to (plus) 2. The plots represent kernel density estimates. Solid horizontal lines denote the median and dashed lines denote the interquartile (25th–75th) range. Overall group differences were assessed using the Kruskal–Wallis test, followed by Dunn’s post hoc test with Holm correction. Pairwise adjusted *p*-values are shown above comparison brackets.

Violin plots (Figure 4) demonstrated right skewed and heterogeneous VAF distributions across all response categories. Although median VAF increased progressively from PR to SD and PD, substantial overlap persisted between individual observations, particularly between SD and PD, consistent with the modest effect sizes and lack of statistical separation after multiple-comparison correction. The broader upper tails observed in the SD and PD distributions reflected a subset of patients with markedly elevated ctDNA burden despite considerable overlap at lower VAF values.

Some patients had additional radiological PD observations during the study screening-recruitment-enrollment phase where plasma specimens were also collected. Thus, the 150 PD measurements included baseline and pre-baseline PD observations. As an additional sensitivity analysis, these baseline and pre-baseline PD observations were excluded, restricting the analysis to 245 longitudinal imaging-concurrent assessments (90 PR, 117 SD, and 38 PD) obtained during follow-up. Frequencies of non-zero measured VAFs remained comparable across response categories (86.7%, 87.2%, and 89.5% for PR, SD, and PD, respectively). ctDNA VAF continued to differ significantly across response groups (Kruskal–Wallis H = 14.08, *p* = 0.000877). Median VAF remained lowest in PR (0.055%; Range 0.0–27.9%; IQR 0.025–0.218%), intermediate in SD (0.190%; Range 0.0–46.7%; IQR 0.040–1.690%), and slightly lower in longitudinal PD assessments than in the complete cohort (0.165%; Range 0.0–59.8%; IQR 0.043–9.125%), while retaining substantially greater variability than PR. Post hoc Dunn’s tests with Holm correction again demonstrated significant differences between PR and SD (adjusted *p* = 0.00375) and between PR and PD (adjusted *p* = 0.00431), whereas SD and PD remained indistinguishable (adjusted *p* = 0.4502). Cliff’s delta effect sizes were highly consistent with the complete-cohort analysis (PR vs. SD, δ = −0.264; SD vs. PD, δ = −0.087; PR vs. PD, δ = −0.336), indicating that exclusion of baseline PD observations did not materially alter the relationship between ctDNA burden and radiographic response.

Because RECIST-defined progressive disease (PD) on the immediately preceding line of therapy was an eligibility criterion for study enrolment, the pooled RECIST-defined stable disease (SD) category was highly asymmetric. Of the 117 SD observations, 112 (95.7%) represented stabilization following baseline PD, whereas only 5 (4.3%) represented maintenance after an initial partial response (PR). Accordingly, the pooled SD category encompassed both persistently elevated ctDNA levels associated with stabilization after prior progression and low ctDNA levels associated with sustained treatment response, contributing to the observed overlap between the SD and PD ctDNA distributions. To explore the influence of this longitudinal heterogeneity, SD observations were subsequently reassigned according to the preceding response trajectory, grouping post-PR SD with PR (5 + 90 = 95) and post-PD SD with PD (112 + 150 = 262). This exploratory re-assignment yielded VAF distributions that remained highly consistent with the primary RECIST-based analysis, with median VAFs of 0.060% (IQR 0.025–0.265%) for the reclassified PR group and 0.190% (IQR 0.040–3.100%) for the reclassified PD group (Mann–Whitney U, *p* = 4.23 × 10^−5^), indicating that the observed molecular separation between response and progression was preserved after longitudinal reclassification.

Collectively, these findings demonstrate that the association between ctDNA burden and radiological response is maintained throughout longitudinal follow-up and is not driven solely by the universally progressive baseline disease state. While ctDNA burden differed significantly across RECIST-defined response categories at the population level, the substantial overlap between individual VAF distributions indicates that single ctDNA measurements should be interpreted within the broader clinical context rather than as deterministic indicators of response state. This observation suggests that the principal value of personalized ctDNA monitoring lies not in isolated VAF measurements but in serial within-patient assessment of molecular dynamics over time. Accordingly, the subsequent analyses evaluated whether longitudinal changes in ctDNA more closely reflected anatomical tumor burden and whether evolving molecular trajectories could identify disease progression before conventional radiological assessment.

### 2.4. ctDNA–Tumor Burden Correlation

**Figure 5:**
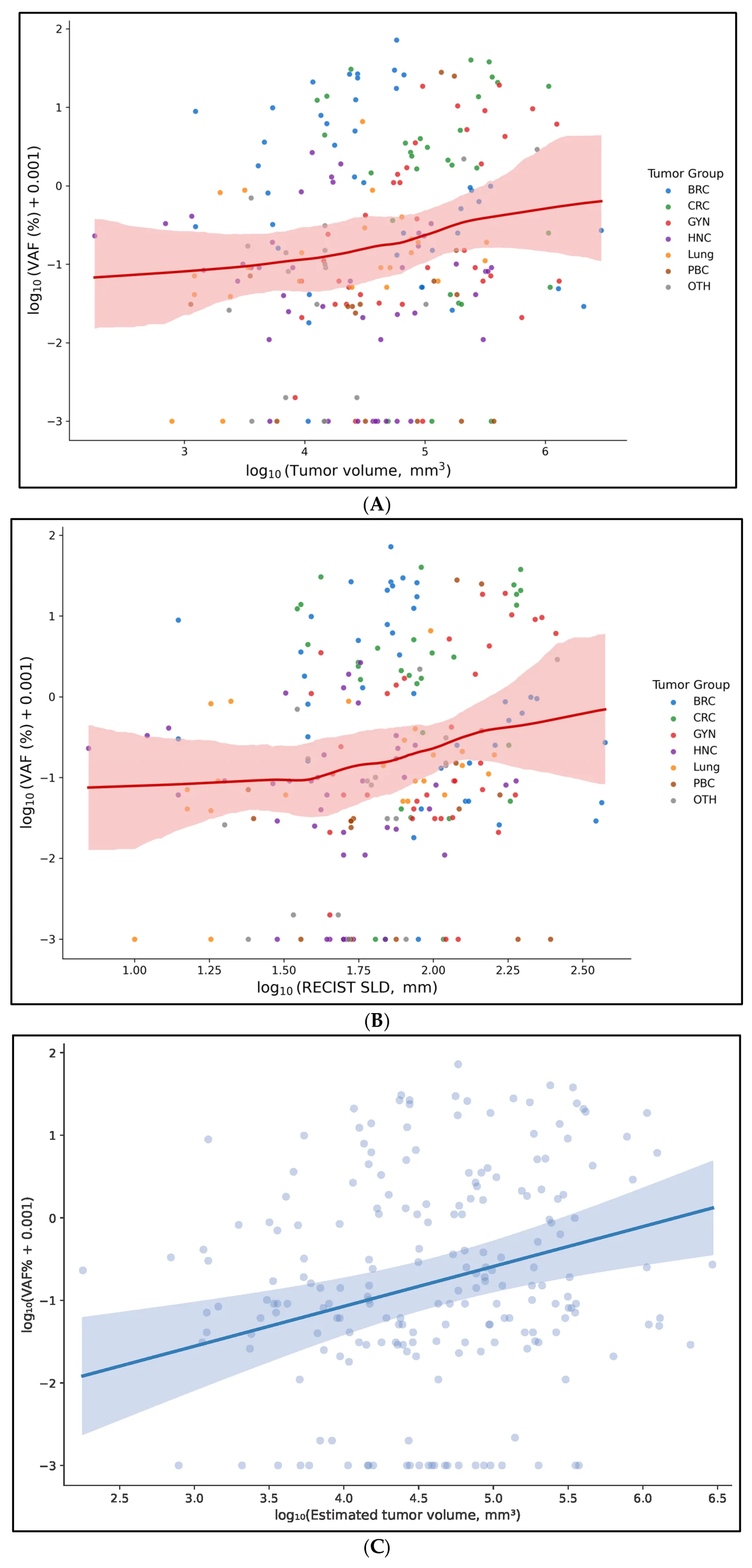

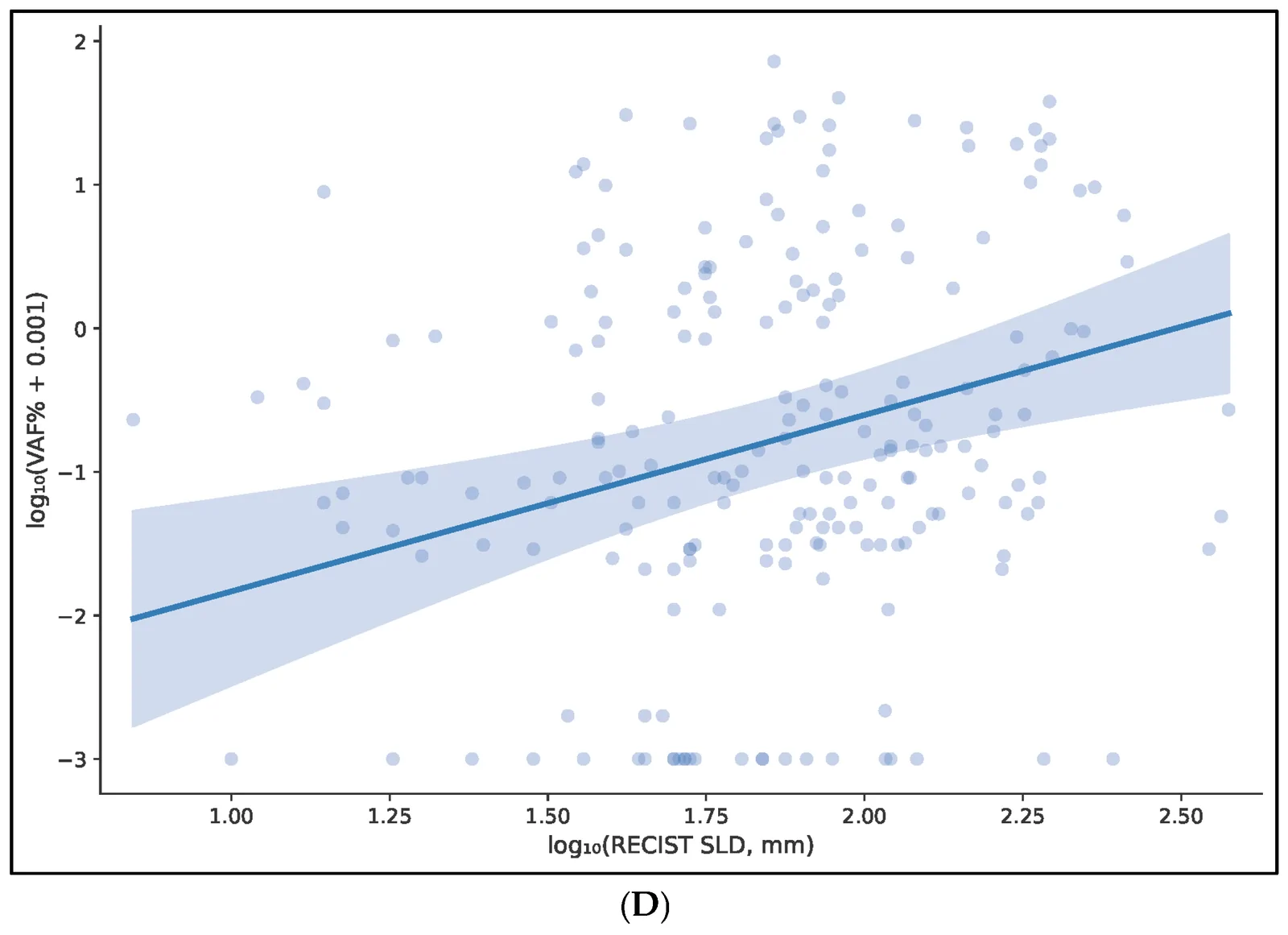
Association between ctDNA burden and radiological tumor burden. The primary analysis comprised 210 imaging–ctDNA pairs obtained within ±7 days from 53 patients. (**A**,**B**) Raw-data scatterplots showing log10(VAF% + 0.001) versus (**A**) log10-transformed estimated radiological tumor volume (mm^3^) and (**B**) log10-transformed RECIST sum of longest diameters (SLD, mm). RECIST SLD is the directly measured reference anatomical metric; estimated volume is an exploratory surrogate reconstructed from available lesion dimensions and includes imputation where orthogonal dimensions were not reported. Each point represents one paired observation and is colored by tumor group: breast (BRC, blue), colorectal (CRC, green), gynecological (GYN, red), head and neck (HNC, violet), lung (orange), pancreaticobiliary (PBC, brown), and other malignancies (OTH, grey). Red curves represent locally estimated scatterplot smoothing (LOESS), with shaded regions indicating 95% confidence intervals (CI). Spearman rank correlations were used as descriptive measures of association. (**C**,**D**) Marginal-effects plots showing the corresponding population-level relationships for (**C**) estimated tumor volume and (**D**) RECIST SLD. Lightly displayed points represent individual paired observations; solid lines represent fixed-effects predictions from linear mixed-effects regression models incorporating patient identifier as a random intercept to account for repeated longitudinal measurements, and shaded regions indicate 95% CI for the estimated marginal mean. Estimated tumor volume was positively associated with ctDNA burden (β = 0.483, 95% CI: 0.216, 0.751; *p* = 0.00040), as was RECIST SLD (β = 1.228, 95% CI: 0.559, 1.898; *p* = 0.00032).

To evaluate the relationship between ctDNA burden and anatomical tumor burden, serial ctDNA measurements were paired with radiological assessments. Of the 61 patients in the clinical analytical cohort, 8 had no evaluable paired radiological tumor-burden measurements and were excluded. The complete dataset comprised 224 paired observations from 53 patients, of which 210 observations were obtained within ±7 days of imaging and constituted the primary Concurrent Group 1 analysis. Fourteen observations had imaging–ctDNA intervals of 8 days or more and constituted Concurrent Group 2. The primary cohort spanned seven tumor groups and included a median of 4 paired observations per patient (range, 1–8). Scatterplots demonstrated weak positive relationships between ctDNA variant allele fraction (VAF) and both estimated tumor volume and RECIST sum of longest diameters (SLD), with substantial variability among observations (Figure 5A,B).

In the primary analysis, descriptive Spearman correlations demonstrated weak but statistically significant positive associations between ctDNA VAF and estimated tumor volume (ρ = 0.169, *p* = 0.0144) and between ctDNA VAF and RECIST SLD (ρ = 0.177, *p* = 0.0101). Because patients contributed repeated longitudinal observations, linear mixed-effects regression models incorporating patient identifier as a random intercept were used for primary inference. Both estimated tumor volume (β = 0.483, 95% CI: 0.216, 0.751; *p* = 0.00040) and RECIST SLD (β = 1.228, 95% CI: 0.559, 1.898; *p* = 0.00032) remained significantly associated with ctDNA burden after accounting for within-patient clustering. The mixed-effects models identified statistically detectable cohort-level associations, but the weak rank correlations (ρ ≈ 0.17) indicate that absolute VAF tracked anatomical tumor burden weakly at the individual-observation level (Table 3).

Exploratory tumor-group analyses demonstrated heterogeneity in both the magnitude and direction of these associations (Supplementary Table S3). Gynecological cancers demonstrated nominally significant positive correlations for both RECIST SLD (ρ = 0.365, *p* = 0.0262) and estimated tumor volume (ρ = 0.484, *p* = 0.0024). Positive correlations with SLD were also observed in the lung (ρ = 0.408, *p* = 0.0479) and heterogeneous other-malignancy groups (ρ = 0.509, *p* = 0.0309). In contrast, breast cancer demonstrated an inverse association between ctDNA VAF and SLD (ρ = −0.480, *p* = 0.0015), although its association with estimated tumor volume was not significant. The remaining subgroup associations were weak or non-significant. Because these analyses involved small patient numbers, repeated observations, and no adjustment for multiple comparisons, they should be regarded as exploratory and hypothesis generating.

The secondary analysis incorporating all 224 observations produced results similar to the primary analysis. Spearman correlations remained weak but statistically significant for estimated tumor volume (ρ = 0.181, *p* = 0.0065) and RECIST SLD (ρ = 0.194, *p* = 0.0035). Corresponding mixed-effects models also remained significant for estimated tumor volume (β = 0.510, 95% CI: 0.246, 0.775; *p* = 0.00016) and RECIST SLD (β = 1.242, 95% CI: 0.583, 1.901; *p* = 0.00022). Thus, inclusion of observations with longer imaging–ctDNA intervals did not materially alter the direction or magnitude of the findings.

Overall, ctDNA burden was positively associated with anatomical tumor burden at the cohort level, but the relationship was weak and varied substantially across tumor groups and individual patients. Individual ctDNA measurements therefore provide only a partial representation of anatomical disease burden. These findings support interpretation of ctDNA within serial, patient-specific molecular trajectories rather than as isolated measurements and provide the rationale for the subsequent analyses of molecular–radiological concordance and molecular progression preceding conventional radiological progression.

### 2.5. Molecular–Radiological Longitudinal Concordance and Molecular Lead Time Analysis

Longitudinal Concordance Level (LCL) framework is explained in Supplementary Table S4—concordance was assessed across the complete molecular and radiological trajectory and included both positive concordance (increasing ctDNA accompanying subsequent or contemporaneous worsening disease) and negative concordance (stable or decreasing ctDNA accompanying sustained response or disease control).

Of the 61 enrolled patients, 12 were excluded because adequate longitudinal on-treatment follow-up was unavailable. The resulting cohort comprised 49 patients contributing 50 evaluable longitudinal trajectories, including two independent treatment episodes from one patient (PID 13770a and 13770b), which were analyzed separately. Fourteen patients subsequently developed RECIST-defined progressive disease following an initial partial response (PR) or stable disease (SD) and comprised the molecular lead-time (MLT) cohort.

Across all 50 evaluable trajectories, 21 (42.0%) demonstrated strong longitudinal concordance (LCL-1), 2 (4.0%) moderate concordance (LCL-2), 11 (22.0%) suggestive concordance (LCL-3), and 16 (32.0%) discordant or non-informative trajectories (LCL-4) (Supplementary Table S5).

Among the 27 patients who achieved RECIST-defined PR (Supplementary Table S6), 18 (66.7%) demonstrated strong longitudinal concordance (LCL-1), 2 (7.4%) moderate concordance (LCL-2), 2 (7.4%) suggestive concordance (LCL-3), and 5 (18.5%) discordant trajectories (LCL-4). Discordant molecular behavior during sustained PR was characterized predominantly by persistent or increasing ctDNA despite continued radiological response. Exploratory comparison showed no apparent reduction in recorded PFS or OS among these five LCL-4 trajectories relative to the LCL-1 PR subgroup; however, the small number of discordant cases, heterogeneous censoring, and post hoc subgrouping preclude meaningful prognostic inference, and these episodes were therefore retained as unresolved molecular–radiological discordance.

The Molecular Evidence Level (MEL) framework for adjudication of molecular lead-time (MLT) events is explained in Supplementary Table S7. Within the MLT cohort (n = 14; Supplementary Table S8), molecular progression preceded radiological progression in 9 patients (64.3%), occurred concurrently with radiological progression in 3 (21.4%; MLT = 0), and no qualifying pre-radiological molecular-progression event was identified in 2 (14.3%). Among the nine positive MLT episodes, five were classified as MEL-1, two as MEL-2, and two as MEL-3. Positive lead times ranged from 28 to 83 days for MEL-1, 50 to 209 days for MEL-2, and 55 to 83 days for MEL-3, with an overall median of 55 days (IQR 46–83; range 28–209). These lead times are presented descriptively because the positive-MLT subset is defined by molecular progression occurring before radiological progression.

Because ctDNA findings remained blinded throughout the study, the potential implications of molecular-leading intervals for clinical reassessment were explored retrospectively. Molecular progression preceded radiological PD in 9 of 14 (64.3%) patients, with positive lead times summarized descriptively. Radiological PD was confirmed at the immediately subsequent assessment in 4 of 9 cases, whereas 5 of 9 had one or more intervening PR or SD assessments after molecular progression before RECIST-defined PD, representing a sustained molecular leading interval during which confirmatory ctDNA testing or earlier imaging could potentially have been considered. Conversely, five PR trajectories (PIDs 14461, 15549, 15623, 16825, and 17733) demonstrated persistent or increasing ctDNA without subsequent radiological progression during available follow-up. Follow-ups from the first documented PR to the last recorded clinical follow-up were 309, 61, 560, 387, and 109 days, respectively (median 309 days; range 61–560). These trajectories were therefore retained as unresolved molecular–radiological discordance rather than classified as MLT events. Available follow-up was also subject to administrative censoring at study closure/data cutoff, and absence of subsequent radiological progression was not interpreted as evidence that later progression could not have occurred.

To determine whether analytical sensitivity influenced longitudinal interpretation, trajectories were stratified according to median effective assay LoD. Strong or moderate concordance was observed in 47.4%, 50.0%, and 41.2% of the low- (≤0.020% VAF), intermediate- (>0.020% to ≤0.0433%), and high-LoD (>0.0433%) groups, respectively, with no evidence of an association between LoD tertile and LCL distribution (permutation *p* = 0.906). Similar findings were obtained using baseline and maximum effective LoD. Nevertheless, LoD-proximal measurements limited interpretation in individual trajectories, particularly those remaining below the assay-specific LoD throughout follow-up.

The complementary LCL and MEL frameworks evaluate different dimensions of longitudinal molecular behavior. LCL summarizes biological concordance across the complete molecular–radiological trajectory, whereas MEL evaluates whether molecular progression temporally precedes radiological progression. Consequently, measurable molecular lead time represents temporally offset (molecular-leading) concordance rather than true biological discordance, because the initial molecular–radiological mismatch is subsequently confirmed by imaging. In contrast, persistent molecular–radiological disagreement without later radiological confirmation weakens evidence for an interpretable molecular-leading interval and is more appropriately classified as discordant or non-informative.

### 2.6. Longitudinal Molecular–Radiological Trajectories

**Figure 6:**
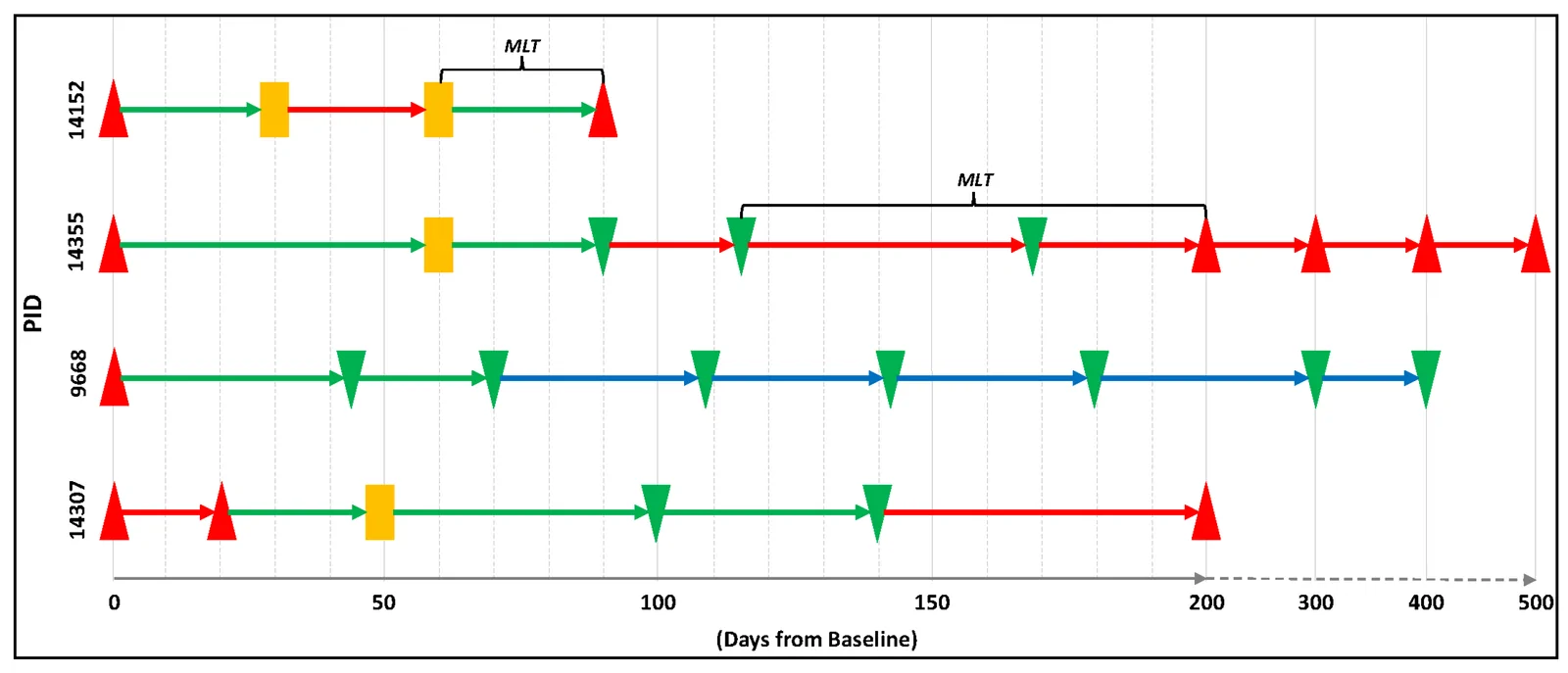
Representative longitudinal molecular and radiological response trajectories demonstrating molecular lead time and longitudinal molecular–radiological concordance. Swimmer-style plots showing the longitudinal trajectories of representative patients. Each segment represents the longitudinal treatment trajectory of one patient and is plotted on the x-axis (days from baseline). The colored arrows indicate the direction (trend, not magnitude) of serial ctDNA change between consecutive time points, with segment color indicating increasing VAF (red), stable VAF (blue), or decreasing VAF (green). Radiological response at each assessment is indicated using symbols: partial response (PR, green downward triangle), stable disease (SD, yellow rectangle), and progressive disease (PD, red upward triangle). The interval between the earliest qualifying molecular progression event and the first radiological evidence of progressive disease (molecular lead time, MLT), where applicable, is indicated. All patients had radiologically confirmed progressive disease (PD) at the baseline (Day 0). PID 14152 (MEL-1): Example of MLT. Following an initial reduction in ctDNA during stable disease (SD), a sustained post-nadir increase in VAF preceded radiological PD by 28 days. PID 14355 (MEL-1): Example of prolonged MLT. ctDNA declined during the initial radiological response (PD→SD→PR), followed by sustained molecular relapse during continued radiological response, preceding radiological PD by 83 days. PID 9668 (LCL-1): Example of molecular–radiological concordance. Rapid reduction of ctDNA to near-background levels was maintained throughout follow-up and remained concordant with sustained radiological response. PID 14307 (LCL-1): Example of molecular–radiological concordance. Progressive reduction in ctDNA accompanied transition from PD to SD and PR. The PD at the final assessment was accompanied by increase in ctDNA. The absence of ctDNA measurements between day 116 and day 180 precluded assessment of whether molecular progression occurred during this interval.

Representative cases were selected to illustrate the spectrum of longitudinal molecular–radiological behavior (Figure 6).

**Figure 7:**
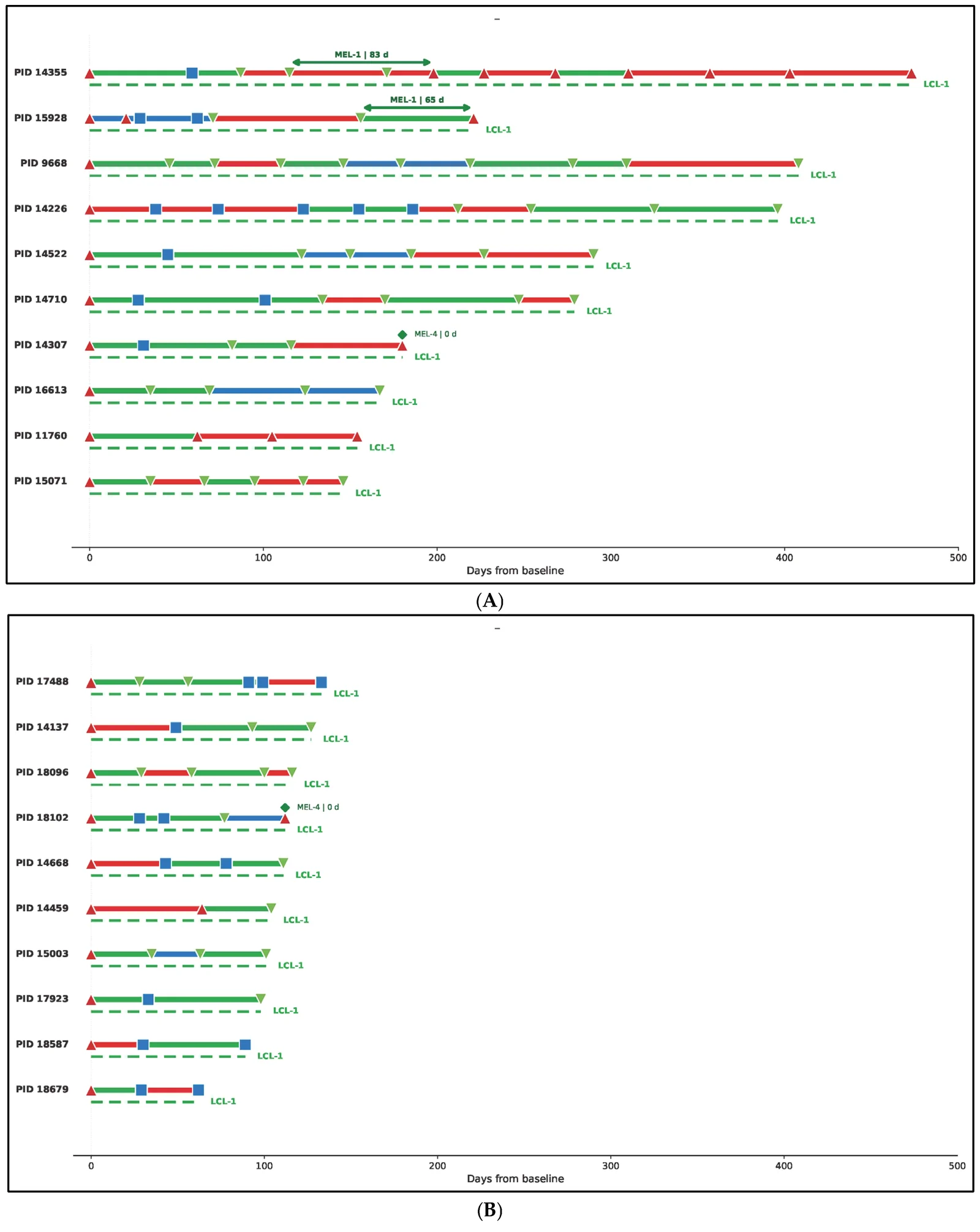

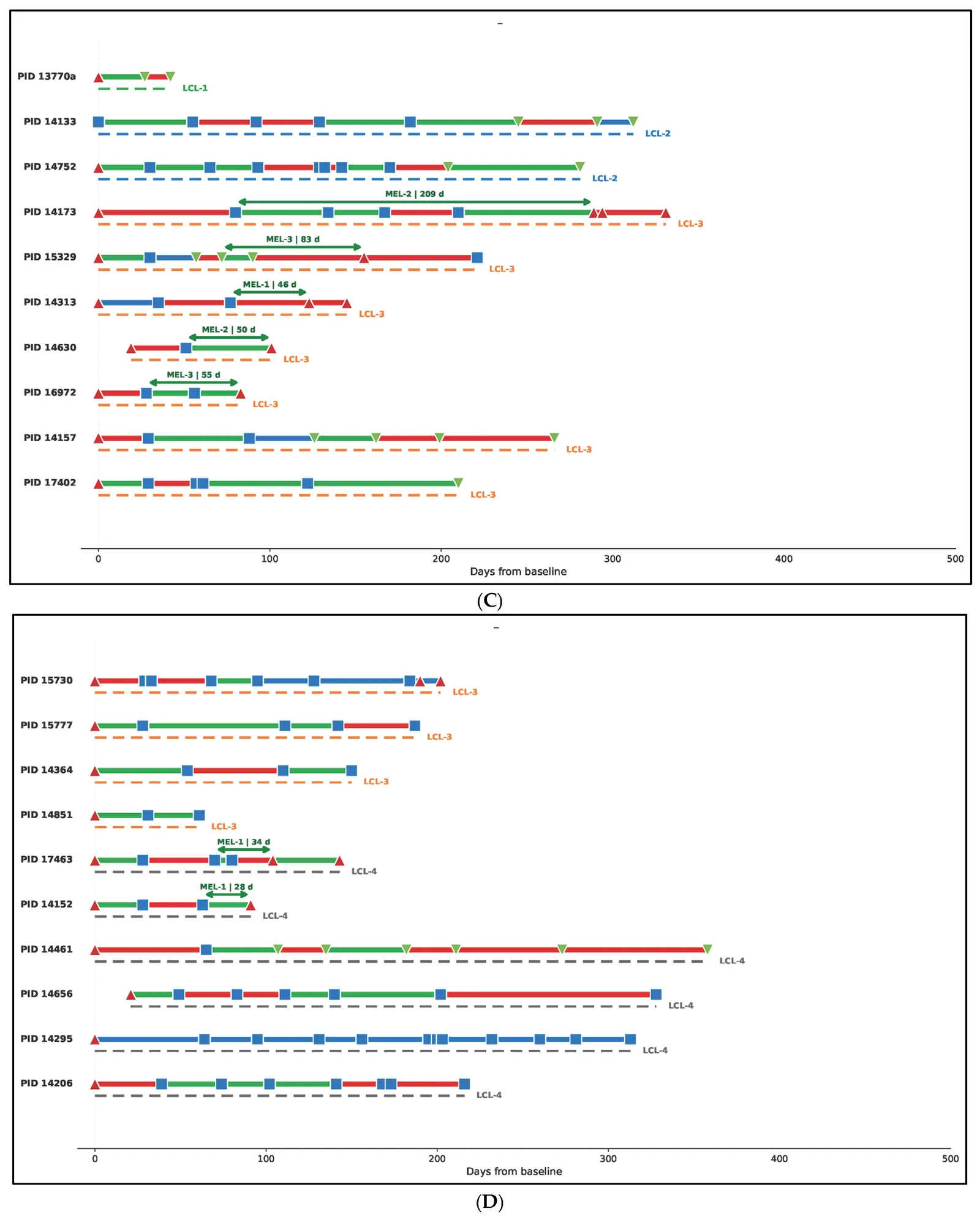

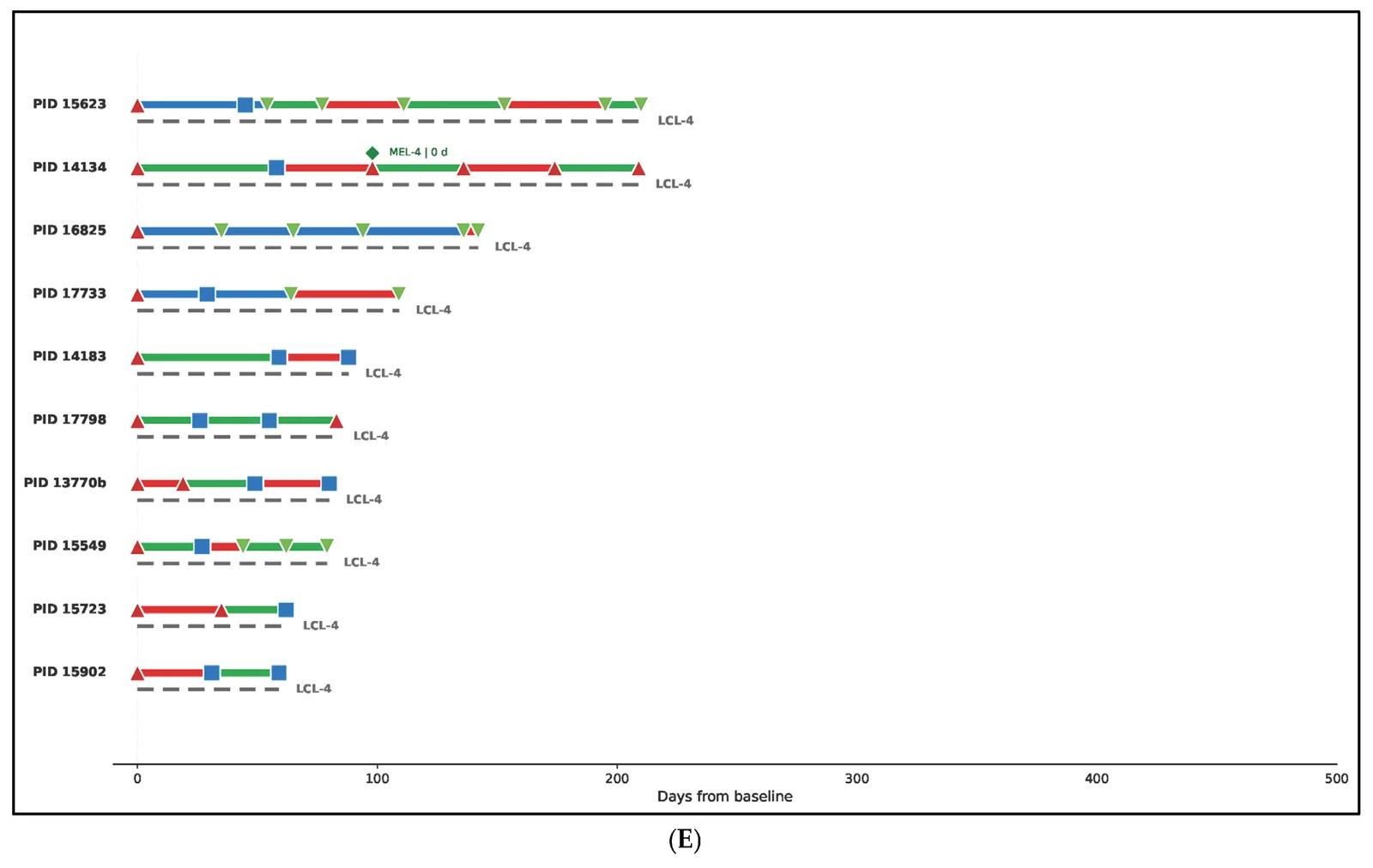
(**A**–**E**) Longitudinal molecular–radiological trajectories across the evaluable longitudinal cohort. Swimmer-style plots showing all 50 evaluable longitudinal trajectories. Panels (**A**–**E**) are display partitions of the same 50-trajectory cohort (10 trajectories per panel) and do not represent distinct analytical subgroups. Each row represents one longitudinal treatment trajectory and is plotted against days from baseline on the x-axis. The central solid line summarizes the direction of serial ctDNA change between consecutive time points, with segment color indicating increasing VAF (red), stable VAF (blue), or decreasing VAF (green). Radiological response at each assessment is superimposed using symbols: partial response (PR, green downward triangle), stable disease (SD, blue square), and progressive disease (PD, red upward triangle). Where applicable, molecular lead-time (MLT) intervals are shown as solid green double-headed arrows, spanning from the earliest qualifying molecular progression signal to subsequent radiological PD; accompanying labels indicate the corresponding Molecular Evidence Level (MEL) and lead time in days. A dashed line beneath each trajectory indicates the overall Longitudinal Concordance Level (LCL) for that trajectory (LCL-1, green; LCL-2, blue; LCL-3, orange; LCL-4, dark grey). These panels illustrate the spectrum of longitudinal molecular–radiological relationships, including durable concordance, temporally offset molecular-leading concordance, unresolved molecular–radiological mismatch, and true discordance.

PID 14355 demonstrated sustained molecular relapse during ongoing PR, preceding RECIST-defined progression by 83 days. PID 14173 showed gradual oscillatory molecular progression that preceded radiological PD by 209 days, illustrating that a measurable molecular-leading interval may also occur in a less uniform longitudinal trajectory. PID 15928 demonstrated molecular clearance followed by ctDNA re-emergence during sustained PR, preceding PD by 65 days. In contrast, PID 9668 illustrated durable negative concordance, with sustained molecular suppression accompanying prolonged PR. PID 16825 demonstrated persistent molecular progression during PR without radiological confirmation during available follow-up, representing unresolved LCL-4 discordance. Finally, PID 17798 showed continued molecular decline despite radiological progression, illustrating true molecular–radiological discordance and reinforcing that ctDNA monitoring complements rather than replaces conventional imaging. Figure 7A–E displays the same 50 evaluable longitudinal trajectories from 49 patients across five readability panels (10 trajectories per panel); panel assignment is graphical only and does not define separate analytical subgroups.

Patient-wise demographic, clinical, and assay details are provided in the Supplementary Dataset. Collectively, these findings demonstrate that serial ctDNA measurements can provide molecular information that is not always synchronous with contemporaneous radiological assessment. In some trajectories, molecular changes preceded subsequent radiological progression, whereas other trajectories remained concordant or discordant. The principal interpretive value of longitudinal ctDNA monitoring in the study therefore lies in distinguishing synchronous concordance, temporally offset concordance, and true molecular–radiological discordance across the complete disease course.

### 2.7. Exploratory Survival Analysis

**Figure 8:**
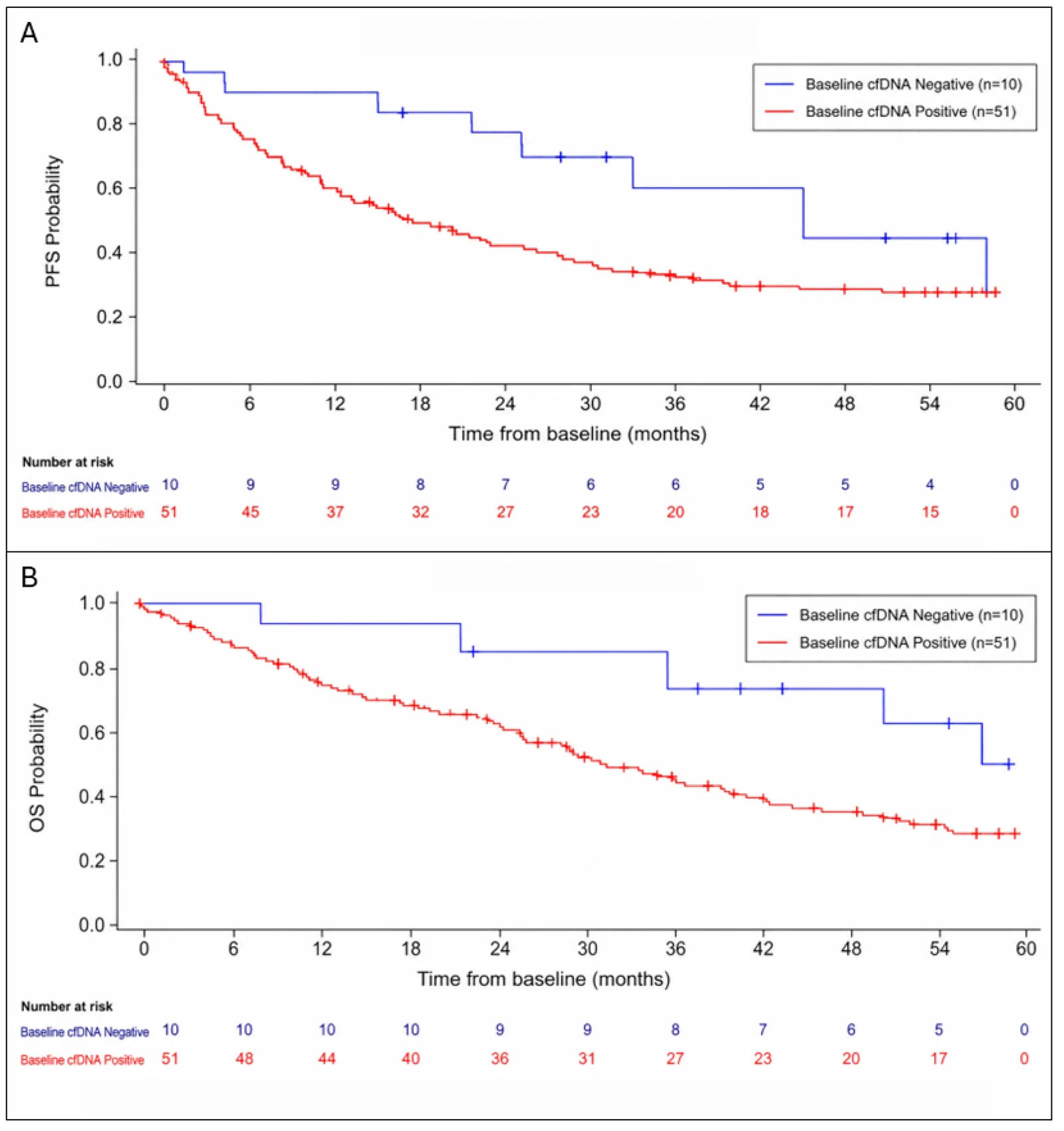
Exploratory survival analyses stratified by baseline ctDNA detection status. (**A**) Progression-Free Survival (PFS) and (**B**) Overall Survival (OS). Survival probabilities were estimated using the Kaplan–Meier method. Tick marks indicate censored observations, and numbers at risk are displayed below each plot. Owing to the heterogeneous pan-tumor design and limited cohort size, these analyses are exploratory and intended to generate hypotheses regarding the prognostic significance of baseline ctDNA detection.

Exploratory Kaplan–Meier analyses were performed in the final evaluable cohort (n = 61) to assess the association between baseline ctDNA detection and clinical outcomes (Figure 8). Patients with detectable baseline ctDNA exhibited shorter progression-free survival (PFS) and overall survival (OS) than ctDNA-negative patients.

Baseline ctDNA positivity was associated with an increased risk of progression or death (HR = 2.38, 95% CI: 0.71, 7.97; log-rank *p* = 0.146) and an increased risk of death (HR = 2.06, 95% CI: 0.48, 8.88; log-rank *p* = 0.322) in unadjusted Cox models. Although Kaplan–Meier curves demonstrated earlier progression and inferior survival among ctDNA-positive patients, neither comparison reached statistical significance. Given the small ctDNA-negative subgroup (n = 10), limited cohort size, and diagnostically heterogeneous pan-tumor population, these findings should be regarded as hypothesis generating rather than definitive evidence of prognostic utility and warrant confirmation in larger prospective studies.

### 2.8. Early Molecular Response and Exploratory Survival Outcomes

In an exploratory landmark analysis restricted to patients with detectable baseline ctDNA and an evaluable post-baseline assessment, a ≥50% VAF reduction by day 60 was associated with directionally lower risks of progression or death (HR 0.53, 95% CI: 0.20, 1.38; *p* = 0.191) and death (HR 0.69, 95% CI: 0.34, 1.42; *p* = 0.315), although neither association reached statistical significance. Similar directional findings were observed using a day-90 landmark for PFS (HR 0.42, 95% CI: 0.13, 1.34) and OS (HR 0.54, 95% CI: 0.25, 1.16). Molecular clearance was uncommon, occurring in five patients by day 60 and six by day 90, and was not amenable to stable Cox-regression analysis because only one subsequent PFS event occurred in each clearance subgroup. These exploratory findings (summarized in Supplementary Table S9) suggest a possible association between early molecular response and improved outcomes but require confirmation in larger, prospectively sampled cohorts. All *p*-values in these exploratory landmark analyses are nominal and were not adjusted for multiplicity; the day-60 clearance PFS comparison is particularly unstable because only five patients achieved clearance and only one subsequent PFS event occurred in that subgroup.

### 2.9. Real-World Feasibility Cohort

To evaluate the operational feasibility and scalability of the personalized tumor-informed ddPCR framework under routine clinical laboratory conditions, a separate real-world cohort comprising 270 patients was analyzed. The cohort had a median age of 55 years (range, 19–91); 204/270 patients (75.6%) were female. Breast cancer constituted the largest tumor group (160/270, 59.3%), with the remaining patients distributed across eight additional tumor groups. A total of 650 personalized ddPCR assays were recorded between 2021 and 2026, corresponding to a median of 2 assay measurements per patient (range, 1–11). At the patient level, 88 of 270 patients (32.6%) underwent two or more serial ctDNA assessments. The remaining 182 patients had a single recorded ctDNA assessment.

All 270 patients had an evaluable baseline assessment, defined as Time Point 1. Because individual patients could have more than one personalized assay at baseline, baseline ctDNA status was determined at the patient level: a patient was classified as baseline-positive if at least one assay performed at Time Point 1 yielded a positive adjudicated ddPCR result. Baseline ctDNA positivity was observed in 88/270 patients (32.6%) (Figure 3).

The assay-level dataset represented 271 distinct gene-variant targets spanning 104 genes. The 650 assay measurements comprised 494 single-nucleotide variant (SNV) assays, 93 insertion/deletion (indel) assays, 53 copy-number amplification (CNA) assays, 7 fusion assays, and 3 promoter mutation assays. The median empirically determined assay-specific limit of detection was 0.020% VAF (IQR, 0.020–0.030%), numerically similar to the median LoD in the clinical cohort. The baseline positivity rate should not be directly compared with that of the clinical cohort because the real-world population differed substantially in clinical composition and was not selected for radiologically progressive advanced disease. Detailed disease burden and clinical indication at the time of testing were not uniformly available; therefore, the observed difference cannot be attributed to a specific clinical setting. Collectively, these data document routine implementation across a broad range of molecular targets and alteration classes within the study sponsor’s laboratory.

## 3. Discussion

This study shows that personalized tumor-informed ddPCR can characterize longitudinal ctDNA dynamics during systemic therapy in patients with advanced solid tumors. Serial ctDNA levels were associated with RECIST response categories at the population level, longitudinal molecular trajectories showed structured concordance and discordance with radiological behavior, and molecular progression preceded radiological progression in a subset of patients who ultimately developed PD. The separate real-world implementation cohort additionally documents operational deployment of personalized ddPCR across diverse tumor types and alteration classes. These observations establish molecular–radiological associations and implementation feasibility; they do not establish the clinical utility of ctDNA-guided treatment modification.

An important finding of this study is that ctDNA behaved as a dynamic longitudinal biomarker rather than a binary indicator of disease. Unlike post-treatment minimal residual disease (MRD) surveillance, where ctDNA positivity primarily reflects the presence or absence of residual disease [27,28], patients with advanced cancer frequently remain ctDNA-positive despite achieving radiological response because viable tumors persist throughout treatment. Consequently, absolute ctDNA positivity is less informative than serial within-patient changes over time. This distinction was further illustrated by the exploratory longitudinal reclassification of RECIST-defined stable disease according to the preceding treatment trajectory. Reassignment of post-response stable disease with PR and post-progression stable disease with PD preserved the principal VAF distributions, supporting the concept that longitudinal biological context provides greater interpretive value than cross-sectional RECIST classification alone.

Consistent with this concept, ctDNA VAF showed population-level association with RECIST response category, with the clearest separation between PR and both SD and PD; SD and PD remained substantially overlapping and were not statistically separated in either descriptive or patient-clustered analyses. Longitudinal trajectories demonstrated structured molecular–radiological concordance in a subset of patients. Individual VAF distributions overlapped substantially, particularly between SD and PD. The longitudinal composition of the SD category, which included both stabilization following prior progression and sustained disease control after response, may have contributed to this overlap. The present analyses do not permit attribution of the overlap to any single biological or analytical factor. The study-specific Longitudinal Concordance Level (LCL) framework further showed that 42% of evaluable trajectories demonstrated strong concordance and that 74.1% of patients achieving partial response exhibited strong or moderate molecular–radiological agreement (20/27; LCL-1, 18/27; LCL-2, 2/27). Collectively, these observations support longitudinal ctDNA kinetics as a complementary molecular correlate of treatment response that complements episodic anatomical imaging rather than replacing it [29]. Together with the tumor burden analyses, these findings indicate that serial ctDNA monitoring provides two distinct but complementary forms of information: confirmation of biological concordance with radiological response, and identification of temporally offset molecular progression that may precede anatomical progression. Accordingly, the principal interpretive value demonstrated in this study lies in serial within-patient molecular trajectories rather than isolated VAF measurements.

A second major finding is the demonstration of measurable molecular lead time before radiological progression. Among patients with evaluable lead times, molecular progression preceded RECIST-defined progression by up to 209 days. Strong MLT events occurred across trajectories with differing overall LCL classifications, reinforcing that MEL and LCL evaluate distinct dimensions of longitudinal molecular–radiological behavior. The complementary LCL and Molecular Evidence Level (MEL) frameworks evaluate different dimensions of longitudinal ctDNA behavior. LCL assesses biological concordance across the complete molecular–radiological trajectory, whereas MEL evaluates whether molecular progression precedes radiological progression. Consequently, measurable molecular lead time does not necessarily represent biological discordance; rather, it reflects a period of temporally offset concordance in which ctDNA anticipates anatomical progression before subsequent radiological confirmation. Conversely, persistent molecular–radiological disagreement without later radiological confirmation represents true biological discordance and weakens evidence supporting an interpretable molecular-leading interval. This distinction recognizes that durable molecular–radiological concordance may exist even in the absence of measurable lead time, and strong lead-time events occurred across trajectories with differing overall LCL classifications, further demonstrating that MEL and LCL capture distinct dimensions of longitudinal behavior. The observed lead times are consistent with previous tumor-specific studies of personalized ctDNA monitoring in metastatic breast cancer and melanoma [30,31,32] while extending these observations to a prospective multi-histology cohort using a unified analytical framework. Given the small number of evaluable progression episodes (n = 14), these MLT observations should be regarded as hypothesis-generating estimates rather than evidence that ctDNA progression should independently trigger treatment modification. These findings quantify opportunities for earlier reassessment rather than proven management benefit. The appropriate response to an isolated molecular signal would be confirmatory sampling and/or earlier imaging, not automatic treatment modification, and clinical utility remains to be tested prospectively.

Structured approaches to interpreting serial ctDNA response have been proposed previously. Jakobsen and Spindler proposed ctDNA-RECIST, which classifies quantitative ctDNA changes relative to measurement confidence intervals alongside conventional RECIST, and the RECIST Working Group has subsequently emphasized the need to standardize sampling times, assay thresholds, and cancer-specific interpretation before plasma ctDNA kinetics can serve as a pan-cancer response biomarker [17,18]. Early ctDNA-RECIST categories have also shown prognostic associations in metastatic pancreatic cancer [33], and a randomized phase II study (NCT06562348) is evaluating ctDNA-RECIST-guided treatment decisions against standard imaging-based management. The present LCL/MEL framework is conceptually different: it was not used to replace RECIST or to direct treatment. LCL describes concordance across the complete molecular–radiological trajectory, whereas MEL grades the strength of evidence that a predefined molecular-progression signal temporally preceded radiological PD. Thus, the present study contributes a descriptive longitudinal adjudication and lead-time framework rather than a validated molecular response criterion. Whether either approach improves outcomes when used to guide management requires prospective interventional testing.

Importantly, ctDNA results were not disclosed to treating clinicians and therefore did not influence treatment decisions. Accordingly, this study demonstrates the existence of a measurable molecular progression window rather than the clinical benefit of intervention based on molecular progression. Whether treatment modification triggered by ctDNA progression improves progression-free survival, overall survival, treatment selection, or quality of life remains to be determined in prospective ctDNA-guided interventional studies [29]. These observations suggest that longitudinal ctDNA monitoring may provide an opportunity for earlier biological recognition of treatment failure before conventional imaging demonstrates anatomical progression, although whether acting on this earlier signal improves clinical outcomes remains unknown. Although ctDNA burden demonstrated statistically significant positive associations with both estimated tumor volume and RECIST SLD, the uniformly weak correlations indicate that anatomical tumor burden alone cannot fully account for the observed variability in circulating ctDNA. Accordingly, statistical significance should not be interpreted as evidence of a strong quantitative relationship between absolute VAF and anatomical tumor burden. RECIST SLD is the more directly interpretable anatomical measure in this dataset; estimated volume is exploratory because orthogonal lesion dimensions were imputed when unavailable. Exploratory subgroup analyses demonstrated marked heterogeneity in both the magnitude and direction of ctDNA-tumor burden relationships across tumor types, consistent with the established influence of tumor vascularity, proliferative activity, metastatic distribution, mechanisms of cell death, and ctDNA clearance on circulating tumor DNA kinetics [34,35]. These findings argue against applying universal quantitative VAF thresholds across malignancies and instead support interpretation of serial within-patient molecular trajectories in the context of tumor biology, disease burden, treatment status, and assay performance [7,29]. Rather than establishing a universal quantitative relationship between ctDNA burden and tumor burden, the present data support a patient-centered approach in which serial changes from an individual’s molecular baseline are likely to provide greater biological and interpretive relevance than absolute VAF thresholds.

Recent studies reinforce the prognostic relevance of on-treatment ctDNA kinetics in defined disease settings. For example, early changes in ctDNA were associated with PFS and OS in advanced ER-positive/HER2-negative breast cancer [33], while ctDNA-RECIST categories were prognostic in metastatic pancreatic cancer [36]. These disease-specific data support prospective evaluation of serial ctDNA dynamics but do not establish a universal pan-cancer response threshold.

An additional feature of this research is the integration of a prospective biomarker study with a separate real-world implementation cohort. The clinical trial cohort evaluated molecular–radiological associations prospectively, whereas the implementation cohort documents sustained operational deployment under routine laboratory practice over five years; it should not be interpreted as an external validation cohort for clinical performance.

The separate real-world implementation cohort provides complementary evidence that personalized ddPCR can be deployed routinely across a broad assay repertoire. Across 270 patients, 650 assays were performed, spanning SNVs, indels, copy-number amplifications, fusions, and promoter variants; 88 patients underwent two or more serial assessments. These data demonstrate sustained operational feasibility and assay-design breadth within the study sponsor’s laboratory environment. They should not be interpreted as external validation of the molecular–radiological associations observed in the clinical cohort, nor as evidence that ctDNA-guided treatment changes improve outcomes. The lower baseline ctDNA positivity in the implementation cohort is also not directly comparable with the clinical cohort because the populations differed in stage, treatment setting, and clinical indication. Accordingly, the real-world data primarily support workflow feasibility and scalability rather than independent clinical-performance validation.

These observations complement emerging evidence supporting broader clinical adoption of ctDNA testing across solid tumors [29].

This study has several strengths. Serial ctDNA measurements were prospectively collected alongside protocol-defined radiological assessments, personalized assays were developed from tumor-specific genomic alterations, and longitudinal analyses were performed using predefined adjudication frameworks applied to a locked dataset. The complementary Longitudinal Concordance Level (LCL) and Molecular Evidence Level (MEL) frameworks provide a structured and transparent approach for interpreting longitudinal ctDNA dynamics beyond conventional response classification or binary ctDNA detection.

This study also has several limitations. The clinical cohort and molecular lead-time subgroup were modest in size, and tumor-specific subgroup analyses were exploratory. The multi-histology design also introduces substantial heterogeneity in ctDNA shedding, disease distribution, treatment exposure, and response kinetics; accordingly, pooled pan-tumor associations should not be interpreted as tumor-agnostic clinical performance estimates, and tumor-specific generalizability requires confirmation in larger disease-specific cohorts. Estimated tumor volume was reconstructed from routine radiological measurements rather than dedicated volumetric segmentation. The personalized single-variant monitoring strategy was optimized for sensitive longitudinal tracking but did not evaluate clonal evolution or emerging resistance mechanisms. Accordingly, an increase in the tracked VAF could reflect expansion or treatment-related selection of that molecular clone rather than a proportional increase in total tumor burden. The subsequent radiological confirmation observed in MLT cases supports temporal molecular–radiological concordance but does not by itself distinguish these biological mechanisms. CHIP-related exclusion affected one of the 62 screened patients; however, matched leukocyte genotyping was targeted rather than universal, and residual uncertainty, therefore, remains for variants in genes that can also arise through clonal hematopoiesis.

Effective LoD varied across longitudinal samples and may have limited interpretation in individual low-shedding trajectories. MLT estimates were additionally constrained by the discrete timing of molecular and radiological assessments; consequently, the observed lead time represents the interval detectable under the study sampling and imaging schedules rather than the exact biological onset of progression. Although no cohort-level association between LoD and LCL classification was detected, LoD-proximal signals remained an important source of uncertainty in selected cases. As an observational biomarker study, ctDNA findings did not influence clinical management, and no independent external statistical analysis or central radiological review was performed, although radiological assessments were blinded to ctDNA results and all longitudinal analyses were performed using predefined algorithms applied to a locked dataset. Prospective validation, particularly in ctDNA-guided interventional trials, will be required to determine whether earlier molecular detection translates into improved clinical outcomes.

## 4. Materials and Methods

### 4.1. Study Design, Oversight and Inclusion Criteria

This study integrates data from two complementary cohorts. The first cohort comprised participant samples and data between 2017 and 2019 from RESILIENT (CTRI/2018/02/011808), a single-center, single-arm, Phase II/III study evaluating personalized systemic therapies in patients with relapsed, refractory advanced solid tumors. The clinical trial was approved by the Institutional Ethics Committees (IECs) of the study sponsor and the study site and conducted in accordance with the Declaration of Helsinki [37] and applicable ethical and regulatory requirements including written informed consent.

All participants provided written informed consent (prior to enrolment) for participation, sample collection and analysis and publication of de-identified findings and data. Serial plasma ctDNA monitoring during the active study phase was a planned secondary study objective—these biomarker findings were neither disclosed to the study investigators nor used to guide study conduct. Clinical trial participants were considered for this secondary study if next-generation sequencing (NGS) of baseline tumor tissue DNA (or plasma cfDNA) identified a somatic gene variant (driver) suitable for patient-specific ddPCR assay design. Exclusion was based on evidence of clonal hematopoiesis of indeterminate potential (CHIP) involving the target variant, defined by a white blood cell (WBC)-to-plasma VAF ratio ≥ 0.5. Additional sub-study specific criteria are described in the corresponding Methods and Results sections. This observational biomarker study is reported in accordance with the STROBE [38] and REMARK [39] reporting guidelines.

A total of 62 patients were screened for inclusion in the biomarker analysis. One patient met the predefined CHIP-risk criteria and was excluded before longitudinal ctDNA monitoring, leaving 61 patients in the final clinical analytical cohort.

The second cohort comprised leftover specimens and data from a separate real-world cohort of patients undergoing routine personalized ctDNA monitoring at the study sponsor between 2021 and 2026. This real-world cohort was used to document routine implementation, assay scalability, and operational breadth of the personalized ddPCR workflow under clinical laboratory conditions. All individuals in this cohort had consented to research use of residual specimens and publication of de-identified data. No restrictions were applied with respect to tumor stage or prior therapy, reflecting routine clinical practice.

The clinical trial cohort was used to evaluate molecular–radiological associations, radiological tumor-burden relationships, molecular lead-time patterns, and exploratory survival outcomes, whereas the separate real-world implementation cohort was used to assess operational feasibility, assay scalability, and ctDNA detection across diverse tumor types and genomic alteration classes. No statistical comparisons were performed between cohorts.

### 4.2. Specimen Collection and Processing

In the clinical trial cohort, serial plasma samples were collected for longitudinal ctDNA monitoring during active study phase. Up to 15 mL of peripheral blood was collected into BD EDTA Vacutainer^®^ tubes (BD Biosciences, San Jose, CA, USA) at baseline, i.e., ≤7 days prior to treatment initiation, as well as prior to each scheduled follow-up (clinical assessment or radiological imaging).

Radiological follow-up was scheduled at approximately days 30, 60, and 90–120, followed by approximately 60-day intervals through day 180 and thereafter at approximately 60–90-day intervals, or earlier/later according to the clinical judgment of the study investigator. Where feasible, the corresponding plasma specimen was collected before the scheduled radiological assessment. Paired molecular–radiological assessment was unavailable when blood collection was precluded by the patient’s clinical condition or when a collected specimen could not be processed because of pre-analytical failure, including agglutination or hemolysis.

Plasma was separated within 3 h of collection by double centrifugation (1600× *g*, 10 min, 4 °C; 16,000× *g*, 10 min, 4 °C) and stored at −80 °C. Cell-free DNA (cfDNA) was extracted from 2–4 mL of plasma using the QIAamp Circulating Nucleic Acid Kit (Qiagen, Hilden, Germany) according to the manufacturer’s instructions. DNA quantity and integrity were assessed using the Qubit dsDNA High-Sensitivity Assay (Thermo Fisher Scientific, Waltham, MA, USA) and the Agilent 2100 Bioanalyzer (Agilent Technologies, Santa Clara, CA, USA). The cfDNA input per ddPCR reaction was ≥20 ng. For response concordance and tumor burden analyses, ctDNA samples collected within ±7 days of radiological imaging were considered classified as imaging concurrent. Samples obtained between (±) 8 and 30 days of radiological imaging were classified as near concurrent and flagged for separate analysis. Samples collected >(±)30 days from imaging were excluded from imaging-concurrent correlation analyses.

### 4.3. Tumor Genomic Profiling

Genomic DNA was extracted from fresh tumor tissue or formalin-fixed paraffin-embedded (FFPE) specimens using the PureLink^®^ Genomic DNA Mini Kit (Thermo Fisher Scientific, USA) or the MagMAX FFPE DNA Isolation Kit (Thermo Fisher Scientific, USA), respectively. Targeted tumor sequencing was performed using the Oncomine™ Comprehensive Assay v3 and Ion AmpliSeq™ Comprehensive Cancer Panel (Thermo Fisher Scientific, USA), covering 452 cancer-associated genes across approximately 1.8 Mb. Libraries were prepared according to the manufacturer’s protocols, processed on the Ion OneTouch™ 2 System, sequenced on the Ion PI™ Chip, and analyzed using Ion Reporter™ Software (v5.10). Baseline plasma cfDNA was profiled using the Oncomine™ Pan-Cancer Cell-Free Assay with unique molecular identifier (UMI)-based error suppression to identify candidate somatic variants for longitudinal monitoring.

### 4.4. Blinding, Data Lock and Analytical Oversight

To minimize analytical bias, radiological response assessments were performed independently by board-certified radiologists who were blinded to all ctDNA results throughout the study. ctDNA results were not disclosed to treating clinicians and therefore did not influence therapeutic decision making or radiological response assessment. Longitudinal molecular analyses were performed only after completion of clinical follow-up using a locked dataset. The molecular progression algorithm, eligibility criteria, and adjudication framework were predefined before longitudinal reconstruction of molecular progression events. Candidate molecular lead-time episodes were independently audited against the locked longitudinal ddPCR dataset to verify concordance between the predefined algorithm and the final adjudicated molecular progression assignments. Statistical analyses were subsequently performed using the final locked analytical dataset.

### 4.5. Patient-Specific ddPCR Assays

For each patient, a single somatic driver variant was selected for longitudinal ddPCR monitoring using a pre-specified prioritization strategy: (i) Tier 1: concordant tumor tissue and plasma hotspot mutations; (ii) Tier 2: high-VAF plasma-exclusive mutations, representing probable metastatic tumor shedding; and (iii) Tier 3: tumor tissue-exclusive clonal driver mutations in patients with absent baseline ctDNA. Custom TaqMan^®^ assays (Thermo Fisher Scientific, USA) were designed for each selected variant (Supplementary Table S10). Assay optimization and analytical verification, including the use of synthetic controls (e.g., CEPH 1347-02) and thermal gradient optimization, were performed prior to clinical application.

Personalized ddPCR assays were performed on the QX200 AutoDG Droplet Digital PCR System (Bio-Rad, Hercules, CA, USA) using dual-labeled FAM/HEX hydrolysis probes targeting the mutant and corresponding wild-type alleles. Each reaction contained a minimum cfDNA input of 20 ng and was partitioned into approximately 20,000 droplets before PCR amplification under assay-specific cycling conditions. Wells were required to contain ≥10,000 accepted droplets to meet predefined quality control criteria; reactions failing this threshold or demonstrating excessive droplet “rain” (>15% of positive partitions) were repeated.

Variant allele fraction (VAF) was calculated using QuantaSoft v1.7.4 software (Bio-Rad, USA) as the proportion of mutant DNA copies (‘copies_mut_’) relative to total, i.e., mutant (‘copies_mut_’) and wild type (‘copies_WT_’) target copies per microliter (μL):

VAF (%) = [(copies_mut_/μL)/(copies_mut_/μL + copies_WT_/μL)] × 100.

For the complementary absolute-quantification analysis, mutant concentration was expressed as mutant copies per mL of plasma using the source ddPCR concentration normalized to the processed plasma volume recorded for the corresponding specimen. VAF remained the prespecified primary metric for longitudinal progression and concordance analyses; mutant copies/mL were evaluated as a complementary sensitivity measure.

The ddPCR assays were validated for limit of blank (LoB) and limit of detection (LoD). Assay results were not available to the study investigator and clinical site team during the prospective monitoring period of the clinical trial. The LoD represented the VAF at which the prespecified replicate-detection acceptance criterion was achieved during analytical validation; it was not an absolute lower boundary below which mutant signal could never be observed. Measurements below the LoD could contain a non-zero estimated VAF, but such values did not meet the replicate-performance criterion defining the validated LoD and were therefore distinguished from formal LoD-based molecular positivity where required. For longitudinal molecular-progression adjudication, LoD was used conservatively as specified in the subsequent Methods section on ‘Molecular–Radiological Longitudinal Concordance and Molecular Lead-Time Analysis’.

### 4.6. CHIP Exclusion Protocol

To minimize false-positive ctDNA detection arising from clonal hematopoiesis of indeterminate potential (CHIP), all candidate tumor-specific tracking variants were first reviewed during assay selection against the COSMIC v98 CHIP catalogue and the gnomAD v4.0 population database. Variants with a population allele frequency > 0.1% or occurring in genes with well-established associations with CHIP (including DNMT3A, TET2, ASXL1, JAK2, and PPM1D) were considered at increased risk of representing clonal hematopoiesis, rather than tumor-derived DNA, and underwent parallel genotyping of matched baseline leukocyte DNA. Variants demonstrating a baseline white blood cell-to-plasma VAF ratio ≥ 0.5 were classified as CHIP derived and excluded from longitudinal monitoring.

Because the tumor-informed ddPCR assays were designed using somatic variants previously identified in matched tumor tissue, leukocyte genotyping was performed as a targeted confirmatory strategy for variants with recognized CHIP potential rather than as universal screening of all tracked variants. Although CHIP-associated mutations have also been reported in genes not classically associated with clonal hematopoiesis (including TP53 and, less commonly, other cancer-associated genes), such events are substantially less frequent and were not routinely screened unless additional evidence suggested possible hematopoietic origin. This approach was adopted to minimize false-positive molecular monitoring while maintaining analytical efficiency for tumor-informed assay development.

Matched baseline leukocyte DNA was tested for the sole candidate variant identified in one patient who met the predefined CHIP-risk criteria. The variant met the predefined WBC-to-plasma VAF ratio criterion for CHIP and was therefore classified as CHIP derived. The patient was excluded from longitudinal monitoring.

### 4.7. Radiological Assessment and Tumor Burden Determination

Radiological response assessments were performed by an independent radiologist at the study site, blinded to all ctDNA VAF results. Imaging was performed per standard-of-care (SoC) practice, using contrast-enhanced CT (chest, abdomen, and pelvis; 5 mm slice thickness) or whole-body FDG-PET-CT where clinically indicated. All scans were reported by board-certified radiologists at the enrolling institution and assigned response categories (complete response, CR; partial response, PR; stable disease, SD; progressive disease, PD) as per RECIST v1.1. Radiological response assessment was blinded to and independent of ctDNA data.

Estimated lesion volume was derived from radiological source reports using an ellipsoid approximation. For lesions reported with three orthogonal dimensions, lesion volume was calculated directly using the ellipsoid formula. For bidimensional RECIST measurements, the third orthogonal diameter (D3) was imputed as the smaller of the two reported diameters. For lesions reported using a single longest diameter only, the two unreported orthogonal diameters (D2 and D3) were each imputed as equal to the reported diameter. Estimated total lesion volume at each imaging assessment was calculated as the sum of all measurable index lesion volumes. Because these estimates were derived from routine radiology reports rather than dedicated three-dimensional image segmentation, estimated lesion volume was considered an exploratory surrogate of anatomical tumor burden rather than a direct volumetric measurement.

### 4.8. RECIST Response Concordance

Differences in ctDNA variant allele fraction (VAF) across RECIST v1.1 response categories—partial response (PR), stable disease (SD), and progressive disease (PD)—were evaluated using the Kruskal–Wallis rank-sum test. Where the global test was significant, pairwise post hoc comparisons were performed using Dunn’s test with Holm correction for multiple testing. Effect sizes were quantified using Cliff’s delta.

To account for repeated assessments within patients, a generalized estimating equation (GEE) model with Gaussian family and exchangeable working correlation was fitted to log10(VAF% + 0.001), with RECIST category as the predictor and patient identifier as the clustering variable. Robust sandwich standard errors were used. The two treatment episodes from PID 13770 were retained as separate trajectories but assigned to the same patient cluster. Pairwise model contrasts were Holm adjusted. The Kruskal–Wallis/Dunn analysis was retained as a descriptive distributional analysis; patient-clustered GEE was used for inferential confirmation.

A complementary sensitivity analysis repeated the descriptive and patient-clustered RECIST comparisons using log10(mutant copies/mL + 1) where absolute mutant concentration was available. VAF remained the prespecified primary quantitative metric.

For this RECIST-stratified descriptive analysis, the reported frequency represented assessments with a non-zero measured VAF (>0%), irrespective of whether the measured value exceeded the assay-specific LoD. This descriptive metric was therefore distinct from the validated LoD and from the qualitative assay-call classification. VAF distributions were visualized using violin plots on a log10-transformed scale [log10(VAF% + 0.001)] to accommodate the highly right-skewed distribution and zero-valued observations. Because radiologically documented progression on the immediately preceding systemic therapy constituted an eligibility criterion for the clinical trial, all patients entered the study with baseline RECIST progressive disease (PD) before commencing protocol-directed treatment. Consequently, baseline PD observations should be distinguished from radiological PD occurring during longitudinal follow-up.

### 4.9. ctDNA–Tumor Burden Association

Associations between ctDNA VAF and radiological tumor burden were evaluated using Spearman’s rank correlation coefficient. Two complementary anatomical measures were evaluated, sum of longest diameters (SLD) as per RECIST, and estimated lesion volume. RECIST SLD served as the reference anatomical metric whereas estimated lesion volume was evaluated as an exploratory surrogate measure. To account for repeated longitudinal measurements within individual patients, linear mixed-effects regression models [40] were fitted using log10-transformed VAF [log10(VAF% + 0.001)] as the dependent variable and log10-transformed radiological burden as the fixed effect, with patient identifier included as a random intercept. The primary analysis included imaging–ctDNA pairs obtained within ±7 days of radiological assessment. Sensitivity analyses incorporating larger imaging–ctDNA intervals were performed to assess the robustness of the findings. Tumor-type subgroup analyses were exploratory, were not adjusted for multiple testing, and are presented as hypothesis generating.

### 4.10. Molecular–Radiological Longitudinal Concordance and Molecular Lead-Time Analysis

Longitudinal concordance was assessed using a structured qualitative adjudication of the complete molecular and radiological trajectory relative to the corresponding radiological trajectory. Molecular trajectories were categorized according to their overall longitudinal pattern (e.g., sustained decline, rise after nadir, molecular re-emergence, persistent molecular positivity, stable low-level ctDNA, oscillatory behavior, molecular clearance, or fluctuations around the assay limit of detection [LoD]). Each longitudinal episode was independently adjudicated according to predefined Longitudinal Concordance Levels (LCL-1 to LCL-4, Supplementary Table S4), representing strong, moderate, suggestive, or absent/discordant molecular–radiological concordance, respectively. LCL assignment was based on the complete longitudinal trajectory rather than individual measurements and incorporated both positive concordance (increasing ctDNA accompanying increasing tumor burden) and negative concordance (stable or decreasing ctDNA accompanying stable or decreasing tumor burden).

Longitudinal trajectory classification was performed by one investigator using the complete available molecular and radiological trajectory and the predefined LCL criteria. Each classification was independently reviewed by a second investigator against the same longitudinal molecular and radiological data. Blinding to radiological information was not performed because the temporal and directional relationship between ctDNA dynamics and radiological response constituted an essential component of the LCL classification itself. Where the initial classification and independent review differed, the trajectory was referred to a third investigator (the corresponding author) for adjudication, and the final classification was assigned by consensus. Formal inter-rater agreement statistics were not calculated; accordingly, the present study does not provide a quantitative estimate of inter-observer reproducibility of the LCL framework.

For MLT analysis, molecular progression (mPD) was determined using a pre-specified patient-level sequential algorithm applied uniformly to the locked longitudinal ddPCR VAF dataset without modification after database lock. No patient-specific modifications to the molecular progression algorithm or adjudication criteria were permitted following data lock. For sequential assessment, the molecular nadir was defined as the lowest VAF observed from the episode baseline through the immediately preceding assessment (i.e., a running nadir, with the episode baseline eligible to serve as the nadir); where identical minimum values occurred at consecutive assessments, the earliest was designated the nadir.

mPD was assigned at the first subsequent assessment demonstrating either (i) a ≥50% increase in VAF above the nadir with the observed VAF exceeding the patient-specific assay LoD, or (ii) transition from a confirmed molecular-negative state (VAF below the assay-specific LoD at two or more consecutive assessments) to a confirmed molecular-positive state. Where additional serial samples were available, persistence or further increase of the molecular signal at the subsequent assessment was required for confirmation; however, the mPD date was assigned to the earliest qualifying assessment to preserve the earliest clinically detectable molecular signal for lead-time estimation. Minor intermittent fluctuations after the nadir were considered compatible with expected biological and analytical variability and did not preclude mPD assignment provided that subsequent serial measurements demonstrated sustained molecular progression. When no subsequent sample was available, the qualifying molecular signal was retained as provisional mPD.

Radiological progression was assigned according to the earliest documented evidence of disease progression using the following hierarchy: (i) RECIST v1.1 progressive disease on contrast-enhanced CT; (ii) progressive disease on whole-body FDG-PET-CT; or (iii) clinically confirmed disease-specific progression at death when terminal imaging was unavailable. Where radiological progression occurred without a contemporaneous ctDNA sample, the radiological progression date remained the reference endpoint and the most recent preceding ctDNA assessment was used for lead-time determination. Molecular lead time (MLT) was calculated as: MLT = Date of Radiological Progression − Date of Molecular Progression. Positive values indicate molecular progression preceding radiological progression, whereas zero indicates simultaneous detection. Negative lead times, representing radiological progression preceding molecular progression, were considered non-informative and were reported descriptively where observed.

Because monitoring was based on a single patient-specific somatic variant, mPD was operationally defined as progression of the tracked molecular signal and was not assumed, in isolation, to establish whole-tumor progression. Potential alternative explanations for an increasing VAF included expansion or treatment-mediated selection of the tracked clone. To reduce overinterpretation of isolated clonal or analytical changes, mPD adjudication incorporated signal magnitude relative to nadir and LoD, persistence on subsequent sampling where available, the complete longitudinal trajectory, and subsequent radiological behavior through the MEL framework.

To complement the algorithmic MLT analysis, each progression episode in the MLT cohort was classified according to predefined Molecular Evidence Levels (MEL-1 to MEL-4, Supplementary Table S7), representing strong, moderate, suggestive, or absent evidence supporting true molecular lead time, respectively. MEL assignment incorporated the temporal relationship between molecular and radiological progression together with the robustness, persistence, and analytical confidence of the molecular trajectory.

MEL classification followed the same adjudication procedure as LCL classification: an initial assignment by one investigator, independent review by a second investigator, and resolution of disagreements by a third investigator (the corresponding author) through consensus adjudication. Because MEL specifically evaluates the temporal relationship between molecular and radiological progression, adjudicators necessarily had access to the relevant radiological trajectory and progression dates. Formal inter-rater agreement statistics were not calculated for MEL classifications.

All candidate lead-time cases underwent structured audit against the locked longitudinal ddPCR dataset. All 14 eligible progression episodes were retained for descriptive MLT classification. Lead-time summary statistics (median, IQR and range) were calculated only for episodes with positive computable MLT. Among patients who developed radiological PD after an initial PR or SD, the episodes were annotated either as positive MLT (molecular progression before radiological progression), concurrent molecular and radiological progression (MLT = 0), or no pre-radiological molecular progression. Median, IQR, and range were calculated among positive MLT episodes. No hypothesis test against zero was performed because restricting the analysis to positive lead times conditions on molecular progression having preceded radiological progression.

LCL and MEL were treated as study-specific descriptive evidence-grading frameworks rather than validated response criteria. They assess different dimensions of a trajectory and are neither hierarchical nor expected to agree: LCL summarizes whole-trajectory molecular–radiological concordance, whereas MEL grades the confidence that a qualifying molecular signal temporally preceded radiological progression.

### 4.11. Exploratory Survival Analysis

Exploratory survival analyses were performed for progression-free survival (PFS) and overall survival (OS) using the Kaplan–Meier method. Survival distributions were compared according to baseline ctDNA detection status using the two-sided log-rank test. Hazard ratios (HRs) and corresponding 95% confidence intervals (CIs) were estimated using separate univariable Cox proportional hazards regression models with baseline ctDNA detection status (positive versus negative) specified as the sole predictor variable. Kaplan–Meier plots display censored observations and numbers at risk over time. Given the diagnostically heterogeneous pan-tumor design, limited cohort size, and imbalance between baseline ctDNA-positive and ctDNA-negative subgroups, the survival analyses were considered exploratory and hypothesis generating.

### 4.12. Exploratory Landmark Analysis of Molecular Response and Survival

To evaluate whether early longitudinal ctDNA response was associated with clinical outcomes, exploratory landmark analyses were performed among patients with detectable ctDNA at baseline and at least one evaluable post-baseline ctDNA assessment available by the prespecified landmark. Patients who experienced the outcome or were censored before the landmark were excluded from the corresponding analysis to reduce immortal-time bias.

The primary landmark was set at 60 days after baseline, with a 90-day landmark analysis performed as a sensitivity analysis. For each patient, the lowest ctDNA variant allele fraction (VAF) observed between baseline and the landmark was identified. Early molecular response was defined as a ≥50% reduction in VAF from baseline to the lowest VAF observed by the landmark. Patients not meeting this criterion were classified as molecular nonresponders.

Molecular clearance was evaluated separately and was defined as achievement of an assay-evaluable molecular-negative result by the landmark, according to the assay-specific ctDNA detection classification recorded in the locked dataset. Because molecular clearance was uncommon, clearance analyses were considered descriptive and exploratory.

Progression-free survival (PFS) and overall survival (OS) were calculated from the landmark rather than from baseline. Patients remaining progression-free or alive at the landmark entered the corresponding risk set, and subsequent events and censoring were evaluated from that time point. Survival distributions were estimated using the Kaplan–Meier method and compared using the two-sided log-rank test. Univariable Cox proportional-hazards regression was used to estimate hazard ratios (HRs) and 95% CIs for molecular responders relative to nonresponders.

Because of the modest number of evaluable patients and outcome events, no multivariable adjustment was performed. Analyses were considered exploratory and hypothesis generating. Caution was applied to molecular-clearance analyses because of the small number of patients achieving clearance and the resulting potential for sparse-data bias and unstable Cox-model estimates. A time-dependent Cox analysis of molecular progression was not performed because molecular progression was adjudicated primarily in patients who subsequently developed radiological progression, resulting in potential outcome-dependent ascertainment and circularity when radiological PFS is used as the endpoint.

### 4.13. Real-World Implementation Cohort

A separate real-world implementation cohort was analyzed to evaluate operational implementation and scalability of the personalized tumor-informed ddPCR workflow under routine clinical laboratory conditions. The cohort comprised routine clinical records spanning 2021–2026, with assay-level ddPCR data available for the corresponding implementation cohort. Patient-level cohort characteristics were derived from the curated clinical cohort database, whereas assay-level analytical metrics were derived from the corresponding ddPCR assay database. Cohort summaries, therefore, distinguish patient-level denominators (number of patients, baseline positivity, and longitudinal follow-up) from assay-level denominators (number and type of ddPCR assays performed). Baseline ctDNA positivity was determined at the patient level using Time Point 1. Where multiple personalized assays were performed at baseline, the patient was classified as positive if at least one assay yielded a positive adjudicated ddPCR result; otherwise, the patient was classified as negative.

### 4.14. Statistical Methods

All statistical analyses were performed using Python [41] (version 3.12) with NumPy (ver. 2.5), Pandas (ver. 3.0), SciPy (ver. 1.18), Statsmodels (ver. 0.14), and Matplotlib (ver 3.11). Statistical significance was assessed using two-sided tests with a significance threshold of *p* < 0.05. Continuous variables are presented as median with interquartile range (IQR) or range, as appropriate, while categorical variables are summarized as counts and percentages. As most quantitative variables exhibited non-normal distributions, non-parametric statistical methods [42] were used where appropriate. No formal adjustment for multiplicity was applied across the primary study endpoints, except for Holm correction for post hoc pairwise comparisons following the Kruskal–Wallis test.

Graphical data visualizations were generated using Matplotlib. Scatterplots incorporated locally weighted scatterplot smoothing (LOESS) with 95% confidence bands for descriptive visualization only and were not used for statistical inference. Kernel density estimation (KDE) violin plots included the median and interquartile range (IQR). Swimmer plots displayed longitudinal ctDNA trends, RECIST response assessments, molecular lead-time intervals, and trajectory-level MEL and LCL classifications. Representative trajectories were generated using MS Excel. All graphical smoothing and density estimates were intended solely to aid visualization and did not influence the underlying statistical analyses.

All analyses were performed using the final locked and adjudicated analytical dataset following completion of predefined eligibility review, quality control verification, and longitudinal adjudication. No exploratory reclassification of patient trajectories was undertaken after completion of the primary analyses.

## 5. Conclusions

Personalized tumor-informed ddPCR provides a feasible approach for longitudinal ctDNA monitoring during systemic therapy in advanced solid tumors. ctDNA levels showed population-level association with RECIST response categories, while substantial overlap between SD and PD and weak correlations with anatomical tumor burden limit the use of isolated quantitative measurements as deterministic response indicators. Longitudinal trajectories provided additional context, and molecular progression preceded radiological progression in 9 of 14 patients who developed PD after an initial PR or SD. These observations support prospective evaluation of serial ctDNA as a trigger for confirmatory sampling or earlier reassessment but do not establish clinical benefit from ctDNA-guided treatment modification. The LCL and MEL frameworks used here are study-specific descriptive constructs and require prospective external validation before they can be considered standardized response or decision criteria. Because the clinical cohort was heterogeneous across tumor types and treatments, these findings should likewise not be interpreted as establishing tumor-agnostic clinical performance. The separate real-world cohort additionally supports the feasibility and molecular breadth of routine implementation within the study laboratory but was not designed as an external validation cohort for molecular–radiological clinical performance.

## Figures and Tables

**Table 1:** Study Cohort Demographics. Demographic and tumor-group characteristics of the clinical study cohort (n = 61) and the separate real-world cohort (n = 270). Age is presented as median (range), while gender and tumor-group distributions are presented as numbers and percentages of patients. Tumor groups reflect the primary malignancy classification used for each cohort; detailed constituent primary sites for grouped categories are provided in the table footnotes.

Characteristic	Clinical Study Cohort	Real World Cohort
Cohort Size	61	270
Age, years		
Median (Range)	49 (24–76)	55 (19–91)
Gender, n (%)		
Female;	32 (52.5%);	204 (75.6%)
Male	29 (47.5%)	66 (24.4%)
Tumor Group, n (%)		
Head and Neck (HNC)	13 (21.3%);	4 (1.5%)
Breast (BRC)	12 (19.7%);	160 (59.1%)
Gynecological (GYN)	9 ^1^ (14.8%);	-
Colorectal (CRC)	7 ^2^ (11.5%);	39 ^3^ (14.4%)
Pancreaticobiliary (PBC)	6 ^4^ (9.8%);	4 ^5^ (1.5%)
Lung	5 (8.2%)	16 (5.9%)
Melanomas (MEL)	-	13 ^6^ (4.8%)
Urological (URO)	-	15 ^7^ (5.5%)
Synchronous Malignancies (SYN)	-	6 ^8^ (2.2%)
Other (OTH)	9 ^9^ (14.8%);	13 ^10^ (4.8%)

*Table Footnotes: ^1^* *Cervix (3), Ovary (4), Vagina (2); ^2^* *Colon (3), Rectum (4); ^3^* *Colon (22), Cecum (2), Rectum (13), Anal (2); ^4^* *Pancreas (3), Ampulla of Vater (2), Gallbladder (1); ^5^* *Pancreas (2), Bile Duct (2); ^6^* *Skin (8), Eye (5); ^7^* *Kidney (10), Renal Pelvis (1), Bladder (4); ^8^* *Breast + Lung (2), Breast + Melanoma (2), Breast + Thyroid (1), Ovary + Lung (1); ^9^* *Prostate (1), Esophagus (1), Stomach (4), Small Bowel (1), Liver (1), Testis (1); ^10^* *Prostate (2), Esophagus (1), Esophagogastric Junction (2), Stomach (3), Small Bowel (1), Brain (1), Appendix (2), Skin Merkel Cell Ca (1).*

**Table 2:** Summary statistics for ctDNA VAF stratified by RECIST response category. The table provides the summary statistics for Figure 4 that depict the distribution of ctDNA variant allele fraction (VAF; log_10_[VAF% + 0.001]) across imaging-concurrent assessments classified as partial response (PR), stable disease (SD), or progressive disease (PD) according to RECIST v1.1. Overall differences among response groups were evaluated using the Kruskal–Wallis test, followed by Dunn’s multiple-comparison test with Holm adjustment for pairwise comparisons.

Metric	Partial Response (PR)	Stable Disease (SD)	Progressive Disease (PD)
Total Observations (n)	90	117	150
Non-zero VAF observations, n (%)	78/90 (86.7%)	102/117 (87.2%)	128/150 (85.3%)
VAF median, range (%)	0.055, 0.0–27.9	0.190, 0.0–46.7	0.205, 0.0–59.8
IQR (Q1, Q3; %)	0.025, 0.218	0.040, 1.690	0.040, 6.775
**Overall Comparison**			
**Test**	**Statistic**	***p*** **-value**	**Conclusion**
Kruskal–Wallis	H = 19.09	0.000072	Significant Difference
Pairwise post hoc comparisons (Dunn’s test with Holm correction)			
**Comparison**	***p*** **-value** **(Holm Adjusted)**	**Cliff’s δ**	**Effect Magnitude, Significance**
PR vs. SD	0.00365	−0.264	Small, Significant
SD vs. PD	0.2608	−0.087	Negligible, Not Significant
PR vs. PD	0.000048	−0.324	Moderate, Significant
Patient-**clustered GEE**			
**Comparison**	**Estimate**	***p*** **-value** **(Holm Adjusted)**	**Interpretation**
Global RECIST-category effect	Wald χ^2^ = 12.60 (df = 2)	0.00183	-
SD vs. PR	β = +0.706 (95% CI: 0.315, 1.096)	0.00119	Higher VAF in SD
PD vs. PR	β = +0.776 (95% CI: 0.305, 1.247)	0.00247	Higher VAF in PD
PD vs. SD	β = +0.070 (95% CI: −0.154, 0.295)	0.539	No evidence of separation

*Table Footnotes: Patient-clustered GEE used log10(VAF% + 0.001) as the dependent variable, RECIST category as the predictor, an exchangeable working correlation, and robust sandwich standard errors. Patient identifier was the clustering variable; the two treatment episodes from PID 13770 were assigned to the same patient cluster. Pairwise GEE contrasts were Holm adjusted.*

**Table 3:** Association between ctDNA burden and radiological tumor burden. The primary analysis included 210 imaging–ctDNA pairs obtained within ±7 days from 53 patients, with a median of 4 paired observations per patient (range, 1–8). Descriptive associations were evaluated using Spearman rank correlation. Primary inference was obtained using linear mixed-effects regression with patient identifier included as a random intercept to account for repeated longitudinal measurements. RECIST sum of longest diameters (SLD) was evaluated as the reference anatomical measure, whereas estimated tumor volume was evaluated as an exploratory surrogate of anatomical tumor burden. Also see Figure 5.

Parameter	SLD (mm)	Volume Estimate (mm^3^)
Spearman correlation (ρ)	0.177	0.169
Spearman *p*-value	0.0101	0.0144
Mixed-effects β (95% CI)	1.228 (0.559, 1.898)	0.483 (0.216, 0.751)
Mixed-effects *p*-value	0.00032	0.00040

*Notes: β (mixed-effects regression coefficient) were estimated using log_10_(VAF% + 0.001) as the dependent variable and log_10_-transformed radiological tumor burden as the fixed effect, with patient identifier included as a random intercept. Spearman coefficients are presented as descriptive measures of monotonic association and do not account for repeated longitudinal observations.*

## Data Availability

All study associated data are included in the Supplementary Dataset.

## Supplementary Materials

The following supporting information can be downloaded at: https://www.mdpi.com/article/10.3390/ijms27188340/s1.

## Abbreviations

The following abbreviations are used in this manuscript:

CI	Confidence interval
CNA	Copy-number amplification
CHIP	Clonal hematopoiesis of indeterminate potential
CR	Complete response
CRC	Colorectal cancer
ctDNA	Circulating tumor DNA
ddPCR	Droplet digital polymerase chain reaction
DNA	Deoxyribonucleic acid
FFPE	Formalin-fixed paraffin-embedded
HR	Hazard ratio
IQR	Interquartile range
KDE	Kernel density estimation
LOESS	Locally estimated scatterplot smoothing
LoD	Limit of detection
MLT	Molecular lead time
MRD	Molecular residual disease
NGS	Next-generation sequencing
OS	Overall survival
PCR	Polymerase chain reaction
PD	Progressive disease
PET	Positron emission tomography
PFS	Progression-free survival
PID	Patient identifier
PR	Partial response
RECIST v1.1	Response Evaluation Criteria in Solid Tumors, version 1.1
SD	Stable disease
SLD	Sum of longest diameters
SNV	Single-nucleotide variant
VAF	Variant allele fraction
LCL	Longitudinal Concordance Level
MEL	Molecular Evidence Level
mPD	Molecular progression
rPD	Radiological progression/radiological progressive disease
BRC	Breast cancer
GYN	Gynecological cancers
HNC	Head and neck cancer
PBC	Pancreaticobiliary cancers
OTH	Other solid tumors/other malignancies
