# Supplementary material for: Tumor-Informed ddPCR for Personalized Longitudinal ctDNA Monitoring in Advanced Solid Tumors: Molecular–Radiological Concordance and Molecular Lead Time
Source: Int J Mol Sci. 2026 Sep 19;27(18):8340. doi: 10.3390/ijms27188340 (PMC13607349; doi:10.3390/ijms27188340)
Supplement: Supplementary file 1 [file ijms-27-08340-s001.zip › 01-02 Supp IJMS 03-Sep-2026.pdf]

Supplementary Table S1. Baseline ctDNA positivity stratified by tracked variant. Percentages are not intended for between-group inferences.

Tracked variant	Positive % (n / N)
AKT1 E17K	100% (1 / 1)
APC L1489fs*18	100% (1 / 1)
BRCA2 R2784W	100% (1 / 1)
CDKN2A Y129*	100% (1 / 1)
EGFR D855N	100% (1 / 1)
EGFR E746-A50del	100% (1 / 1)
EGFR L858R	0% (0 / 1)
EGFR T790M	100% (1 / 1)
FGFR3 S249C	0% (0 / 2)
GNAS R201C	100% (2 / 2)
GNAS R201H	100% (2 / 2)
GNAS R844H	100% (1 / 1)
IDH1 R132H	0% (0 / 1)
KRAS G12A	100% (2 / 2)
KRAS G12V	75.0% (3 / 4)
KRAS G13D	100% (1 / 1)
NOTCH1 G1704R	100% (1 / 1)
NRAS G12D	100% (1 / 1)
NRAS Q61L	100% (1 / 1)
PIK3CA E542K	50.0% (1 / 2)
PIK3CA E545K	66.7% (2 / 3)
PIK3CA H1047L	100% (1 / 1)
PIK3CA H1047R	100% (7 / 7)
PIK3CA K111E	100% (1 / 1)
TP53 G266V	0% (0 / 1)
TP53 I254N	100% (1 / 1)
TP53 K132E	50.0% (1 / 2)
TP53 K132R	100% (1 / 1)
TP53 R156P	100% (1 / 1)
TP53 R175H	75.0% (3 / 4)
TP53 R248Q	100% (2 / 2)
TP53 R248W	100% (3 / 3)
TP53 R249S	100% (1 / 1)
TP53 R273H	100% (3 / 3)
TP53 R282W	100% (1 / 1)
TP53 R342P	100% (1 / 1)

Supplementary Table S2. Absolute mutant copies per mL across RECIST response categories as a complementary quantitative analysis. Absolute mutant concentration was available for all 357 imaging-concurrent assessments. Descriptive summaries are shown together with a patient-clustered generalized estimating equation (GEE) sensitivity analysis using log10(mutant copies / mL + 1). VAF remained the prespecified primary quantitative metric because the longitudinal molecular-progression algorithms and assay-specific LoD framework were defined in VAF; mutant copies / mL is presented as a complementary absolute-quantification measure. The GEE model accounted for repeated observations within patients using patient identifier as the clustering variable; the two treatment episodes from PID 13770 were assigned to the same patient cluster. Pairwise GEE contrasts were Holm-adjusted.

Metric	PR	SD	PD
Total observations	90	117	150
Mutant copies / mL			
Median	5.5	13.2	16.1
Range	0–8300	0–7800	0–13000
IQR	1.8–16.1	2.6–104.0	2.05–197.0
Non-zero observations, n (%)	74 (82.2%)	102 (87.2%)	128 (85.3%)

Patient-clustered GEE on log10(copies / mL + 1)	β (95% CI)	Holm-adjusted p	Interpretation
Global RECIST-category effect	Wald χ^2=11.31 (df=2)	0.00349	—
SD vs PR	+0.506 (0.190, 0.822)	0.00336	Higher absolute mutant concentration in SD
PD vs PR	+0.595 (0.241, 0.948)	0.00290	Higher absolute mutant concentration in PD
PD vs SD	+0.089 (−0.100, 0.277)	0.358	No evidence of separation

Supplementary Table S3. Tumor-type stratified associations between ctDNA burden and radiologic tumor volume. Spearman rank correlation analysis between ctDNA variant allele fraction (VAF) and radiological tumor burden by tumor type. Correlation coefficients (ρ) were calculated using imaging–ctDNA pairs obtained within ±7 days. Positive values indicate increasing ctDNA burden with increasing tumor burden, whereas negative values indicate an inverse association. No adjustment for multiple comparisons was performed. Subgroup analyses were exploratory and should not be interpreted as confirmatory owing to limited patient numbers. OTH, other solid tumors; GYN, gynecological cancers; Lung, lung cancer; CRC, colorectal cancer; HNC, head and neck cancer; PBC, pancreaticobiliary cancers; BRC, breast cancer. Statistical significance was defined as $p < 0.05$.

Tumor Type	Patients (n)	Observations	Spearman ρ		p-value	
			VAF vs volume	VAF vs SLD	VAF vs volume	VAF vs SLD
BRC	10	41	−0.229	−0.480	0.1497	0.0015
CRC	7	30	0.012	0.103	0.9488	0.5877
GYN	9	37	0.484	0.365	0.0024	0.0262
HNC	12	44	−0.182	−0.057	0.2382	0.7137
Lung	4	24	0.351	0.408	0.0929	0.0479
PBC	5	16	0.043	0.072	0.8733	0.7902
OTH	6	18	0.274	0.509	0.2704	0.0309

Supplementary Table S4. Longitudinal Concordance Level (LCL) framework for adjudication of serial molecular–radiological trajectories. Definitions of the four Longitudinal Concordance Levels (LCL-1 to LCL-4) used to classify the strength of agreement between serial ctDNA dynamics and corresponding longitudinal radiological tumor behavior. The framework incorporates both positive concordance (increasing ctDNA accompanying worsening disease) and negative concordance (stable or decreasing ctDNA accompanying stable disease or treatment response) and was applied to all evaluable longitudinal trajectories independently of the molecular progression algorithm.

LCL	Concordance	Definition	Typical molecular behavior	Typical radiological behavior	Interpretation	Relationship to the molecular progression algorithm
LCL-1	Strong	Serial molecular and radiological trajectories demonstrate sustained, internally consistent concordance over multiple assessments, providing compelling evidence that ctDNA faithfully reflects longitudinal tumor behavior. Concordance may be either **positive** (increasing VAF accompanying increasing tumor burden) or **negative** (stable / decreasing VAF accompanying stable or decreasing tumor burden).	Sustained increase after nadir preceding or accompanying radiological deterioration, **or** sustained suppression / decrease accompanying durable PR or SD.	PR→SD, SD→PD, PR→PD, or durable PR / SD with continued disease control.	Strong evidence that serial ctDNA accurately tracks longitudinal tumor dynamics.	Descriptively consistent with the predefined molecular-progression algorithm and sustained molecular–radiological concordance.
LCL-2	Moderate	Molecular and radiological trajectories remain generally concordant, but interpretation is tempered by biological or analytical factors such as persistent residual ctDNA, delayed molecular response, treatment-related ctDNA release, limited sampling, or modest fluctuations that do not alter the overall trend.	Persistent positive ctDNA with stable trend, gradual decline, modest sustained increase, molecular persistence, or stable post-treatment ctDNA.	Persistent SD, improving PR, slowly evolving radiological response, or prolonged stable disease.	Moderate evidence that molecular behavior remains biologically concordant with tumor behavior despite one or more limiting factors.	Generally consistent with the predefined algorithm, although the trajectory does not necessarily demonstrate molecular lead time
LCL-3	Suggestive	Molecular trajectory is biologically suggestive of longitudinal concordance but remains speculative because the trend is incompletely established, radiological confirmation has not yet occurred, or alternative explanations cannot be excluded.	Emerging increase after nadir, intermittent rise, possible molecular relapse, incomplete confirmation, or evolving trend.	Stable PR / SD without subsequent documented deterioration, limited follow-up after apparent molecular change, or evolving response.	Suggestive but unconfirmed evidence of longitudinal concordance requiring additional longitudinal observation.	Provides suggestive, hypothesis-generating concordance information but does not independently validate the algorithm.
LCL-4	Discordant / Non-informative	No reproducible longitudinal concordance is demonstrated, or insufficient information exists to establish a meaningful molecular–radiological relationship.	Isolated fluctuations around the LoD, inconsistent VAF behavior, absence of a	Radiological behavior not mirrored by molecular dynamics, persistent PD without	No meaningful evidence of longitudinal concordance.	Does not demonstrate informative longitudinal molecular–

LCL	Concordance	Definition	Typical molecular behavior	Typical radiological behavior	Interpretation	Relationship to the molecular progression algorithm
			reproducible trend, or inadequate follow-up.	evaluable transition, or insufficient longitudinal assessment.		radiological concordance for that episode.

Supplementary Table S5. Patient-level longitudinal concordance analysis of serial ctDNA and radiological response trajectories. Patient-level adjudication of all evaluable longitudinal trajectories using the Longitudinal Concordance Level (LCL) framework. Each longitudinal episode was independently assessed according to the overall concordance between serial ctDNA variant allele fraction (VAF) dynamics and the corresponding RECIST-defined radiological trajectory across the entire available follow-up. Molecular trends, longitudinal interpretations, and final LCL assignments are presented for each trajectory. One patient (PID 13770) contributed two independent treatment episodes (13770a and 13770b), which were analyzed separately as independent longitudinal trajectories.

LCL	PID	RECIST trajectory	Preliminary interpretation
**LCL-1**	9668	PD→PR	Durable molecular suppression throughout sustained PR.
**LCL-1**	11760	PD	Sustained high ctDNA accompanied persistent PD.
**LCL-1**	13770a	PD→PR	Rapid molecular response mirrored durable PR.
**LCL-1**	14137	PD→SD→PR	Progressive molecular decline accompanied radiological response.
**LCL-1**	14307	PD→SD→PR→PD	Excellent molecular response during PR with subsequent concurrent relapse at PD. Overall trajectory highly concordant.
**LCL-1**	14355	PD→SD→PR→PD	Molecular response followed by sustained molecular relapse accompanying disease progression.
**LCL-1**	14459	PD→PR	Molecular clearance accompanied radiological response.
**LCL-1**	14522	PD→SD→PR	Durable molecular suppression accompanied sustained PR.
**LCL-1**	14668	PD→SD→PR	Progressive ctDNA decline mirrored tumor response.
**LCL-1**	14710	PD→SD→PR	Sustained molecular response with only late minor fluctuation.
**LCL-1**	15003	PD→PR	Durable reduction to minimal ctDNA during PR.
**LCL-1**	15071	PD→PR	Sustained molecular suppression throughout PR.
**LCL-1**	15928	PD→SD→PR→PD	Molecular clearance during response followed by molecular relapse before PD. Overall trajectory strongly concordant.
**LCL-1**	16613	PD→PR	Complete molecular response accompanied durable PR.
**LCL-1**	17488	PD→PR→SD	Molecular dynamics remained concordant throughout disease control.
**LCL-1**	17923	PD→SD→PR	Progressive molecular decline mirrored radiological improvement.
**LCL-1**	18096	PD→PR	Sustained low ctDNA despite minor transient fluctuation.
**LCL-1**	18102	PD→SD→PR→PD	Molecular response closely paralleled radiological response; concurrent PD without loss of overall concordance.
**LCL-1**	18587	PD→SD	Persistent increase in ctDNA accompanied persistent disease burden.
**LCL-1**	18679	PD→SD	Stable / increasing ctDNA paralleled persistent disease.
**LCL-1**	14226	PD→SD→PR	Early fluctuations followed by response-associated decline and later low-level variability.
**LCL-2**	14133	SD→PR	Early molecular variability followed by durable molecular response.
**LCL-2**	14752	PD→SD→PR	Overall declining molecular trend despite intermittent fluctuations.
**LCL-3**	14157	SD→PR	Response-associated molecular decline with later emerging increase requiring further follow-up.
**LCL-3**	14173	PD→SD→PD	Persistent molecular evolution broadly consistent with eventual progression but without strong concordance.
**LCL-3**	14313	PD→SD→PD	Molecular increase broadly paralleled subsequent progression.
**LCL-3**	14364	PD→SD	Overall declining molecular trend during persistent SD.
**LCL-3**	15329	PD→SD→PR→PD→SD	Borderline LoD-associated fluctuations with biologically plausible longitudinal concordance.
**LCL-3**	15730	PD→SD→PD	Persistent low-level fluctuations preceding progression.
**LCL-3**	16972	PD→SD→PD	Persistent ctDNA with modest increase before PD.
**LCL-3**	17402	PD→SD→PR	Overall declining but fluctuating ctDNA accompanied improving disease.
**LCL-3**	14630	PD→SD→PD	Limited sampling with molecular behavior broadly compatible with progression.

LCL	PID	RECIST trajectory	Preliminary interpretation
**LCL-3**	14851	PD→SD	Rapid molecular decline accompanying stable disease.
**LCL-3**	15777	PD→SD	Sustained low-level decline during SD.
**LCL-4**	13770b	PD→SD	Fluctuating ctDNA without reproducible longitudinal pattern.
**LCL-4**	14152	PD→SD→PD	Early molecular decline despite persistent disease burden before progression.
**LCL-4**	14183	PD→SD	Variable ctDNA with insufficient longitudinal evidence.
**LCL-4**	14206	PD→SD	Persistent high ctDNA without informative longitudinal evolution.
**LCL-4**	14295	PD→SD	ctDNA remained below LoD throughout persistent SD.
**LCL-4**	14461	PD→SD→PR	Persistent / increasing ctDNA despite durable PR.
**LCL-4**	14656	PD→SD	Marked ctDNA fluctuations without reproducible trend.
**LCL-4**	15549	PD→SD→PR	Persistently high ctDNA despite radiological response.
**LCL-4**	15623	PD→SD→PR	Increasing ctDNA during sustained PR.
**LCL-4**	15723	PD→SD	Fluctuating ctDNA without consistent relationship to disease status.
**LCL-4**	15902	PD→SD	Minimal fluctuations around the LoD with limited interpretability.
**LCL-4**	16825	PD→PR	Progressive molecular increase despite durable PR.
**LCL-4**	17463	PD→SD→PD	Inconsistent molecular behavior with only transient increases.
**LCL-4**	17733	PD→SD→PR	Molecular increase during PR without established longitudinal concordance.
**LCL-4**	17798	PD→SD→PD	Continued molecular decline despite radiological progression.
**LCL-4**	14134	PD→SD→PD	Reducing trend despite radiological progression.

Supplementary Table S6. Patient-level longitudinal concordance analysis in patients achieving partial response (PR). Patient-level adjudication of longitudinal molecular–radiological concordance in patients who achieved a RECIST-defined partial response during follow-up. Each serial trajectory was independently evaluated using the Longitudinal Concordance Level (LCL) framework according to the overall agreement between serial ctDNA variant allele fraction (VAF) dynamics and the corresponding radiological response. The final LCL assignments shown here are the same whole-trajectory classifications reported in Supplementary Table S5; the molecular-trajectory descriptions in this table emphasize behavior during the PR phase to contextualize response-associated concordance and discordance. Preliminary longitudinal interpretations and final LCL assignments are presented for each evaluable trajectory.

LCL	PID	RECIST trajectory	Molecular trajectory	Preliminary interpretation
**LCL-1**	9668	PD, PR	Rapid decline followed by persistent near-LoD ctDNA	Concordant molecular response throughout sustained PR.
**LCL-1**	13770a	PD, PR	Rapid decline to low-level ctDNA	Molecular response paralleled durable PR.
**LCL-1**	14137	PD, SD, PR	Marked reduction during response	ctDNA closely mirrored radiological improvement.
**LCL-1**	14226	PD, SD, PR	Initial fluctuations followed by marked decline with PR	Overall molecular trajectory paralleled radiological response despite early variability.
**LCL-1**	14459	PD, PR	Molecular clearance accompanying PR	Complete molecular response accompanied radiological response.
**LCL-1**	14522	PD, SD, PR	Progressive decline to molecular negativity during PR	Durable molecular suppression accompanied sustained response.
**LCL-1**	14668	PD, SD, PR	Progressive molecular decline	ctDNA closely reflected improving disease burden.
**LCL-1**	14710	PD, SD, PR	Progressive reduction with low residual ctDNA	Sustained molecular response during PR.
**LCL-1**	15003	PD, PR	Rapid reduction to very low ctDNA	Durable molecular suppression.
**LCL-1**	15071	PD, PR	Rapid decline with sustained low ctDNA	Stable molecular response throughout PR.
**LCL-1**	16613	PD, PR	Molecular clearance maintained	Excellent concordance with durable PR.
**LCL-1**	17488	PD, PR, SD	Sustained decline accompanying disease control	Molecular trajectory remained concordant throughout response / stable disease.
**LCL-1**	17923	PD, SD, PR	Progressive decline	Molecular behavior mirrored radiological improvement.
**LCL-1**	18096	PD, PR	Overall decline despite transient fluctuation	Sustained response with continued low ctDNA.
**LCL-1**	18102	PD, SD, PR	Progressive decline to molecular negativity	Excellent molecular response before later progression.
**LCL-1**	14307	PD, SD, PR	Excellent response followed by molecular increase immediately before PD	Molecular response concordant during PR, but limited longitudinal confirmation of relapse before progression.
**LCL-1**	14355	PD, SD, PR, PD	VAF decline accompanying disease stabilization and response, followed by visible MLT and increase with concurrent PD	Molecular response concordance with initial response, followed by a molecular-leading interval preceding radiological PD.
**LCL-1**	15928	PD, SD, PR, PD	VAF decline accompanying disease stabilization and response, followed by visible MLT	Molecular response concordance with initial response, followed by molecular re-emergence constituting a measurable molecular-leading interval before radiological PD
**LCL-2**	14133	SD, PR	Fluctuating during SD followed by molecular response	Early variability but overall molecular trend consistent with eventual PR.
**LCL-2**	14752	PD, SD, PR	Gradual decline with intermittent fluctuations	Overall decreasing trend despite moderate variability.
**LCL-3**	14157	SD, PR	Early variability with later molecular rise during PR	Concordant response initially but emerging molecular change warrants observation.
**LCL-3**	15329	PD, SD, PR, PD	LoD-associated fluctuations after response	Molecular behavior compatible with evolving relapse but analytically borderline.

LCL	PID	RECIST trajectory	Molecular trajectory	Preliminary interpretation
**LCL-4**	14461	PD, SD, PR	Persistent / increasing ctDNA despite sustained PR	Molecular trajectory discordant with radiological response.
**LCL-4**	15549	PD, SD, PR	Persistently high ctDNA with only partial decline	Persistent molecular burden despite radiological response.
**LCL-4**	15623	PD, SD, PR	Increasing ctDNA during sustained PR	Molecular trend inconsistent with radiological improvement.
**LCL-4**	16825	PD, PR	Progressive ctDNA increase despite persistent PR	Discordant molecular behavior (possible occult disease).
**LCL-4**	17733	PD, SD, PR	Progressive increase from molecular negativity	Discordant trajectory with limited longitudinal evidence.

Supplementary Table S7. Molecular Evidence Level (MEL) framework for adjudication of molecular lead-time events. Definitions of the four Molecular Evidence Levels (MEL-1 to MEL-4) used to classify the strength of evidence supporting molecular progression preceding RECIST-defined radiological progression. The framework incorporates the temporal relationship between molecular and radiological progression together with the robustness, persistence, and analytical confidence of the molecular trajectory, and was applied to all evaluable progression episodes within the molecular lead-time cohort.

MEL	Level of Evidence	Definition	Typical molecular behavior	Typical radiological behavior	Interpretation	Relationship to the molecular progression algorithm
MEL-1	Strong	Molecular progression is clearly established before radiological progression by a sustained post-nadir increase (or confirmed molecular re-emergence) that is biologically coherent, analytically robust, and temporally precedes RECIST-defined PD.	Sustained increase after nadir exceeding the assay LoD, usually confirmed on subsequent assessment, with minimal ambiguity.	SD or PR followed by PD after a measurable molecular interval.	Strong evidence that ctDNA identified progression before imaging, providing a reliable estimate of molecular lead time.	Provides strong descriptive evidence that the predefined molecular-progression signal preceded radiological PD in that episode.
MEL-2	Moderate	Molecular progression is biologically plausible and precedes PD but interpretation is limited by one or more factors such as sparse sampling, delayed confirmation, persistent residual ctDNA, borderline analytical behavior, or modest trajectory ambiguity.	Increase after nadir or molecular re-emergence preceding PD, but with incomplete confirmation or limited longitudinal resolution.	SD / PR followed by PD, with plausible but less definitive molecular lead time.	Moderate evidence supporting molecular lead time despite minor analytical or biological limitations.	Provides moderate descriptive evidence of molecular progression preceding radiological PD, with specified analytical or longitudinal limitations.
MEL-3	Suggestive	Molecular findings are compatible with possible molecular progression but do not provide convincing evidence of measurable lead time because the trajectory is incomplete, inconsistent, or only weakly precedes radiological progression.	Intermittent increase, LoD-associated fluctuations, transient molecular positivity, or incompletely established trend.	PD eventually occurs, but temporal precedence of molecular progression remains uncertain.	Suggestive but unconfirmed evidence of molecular lead time.	Provides suggestive, hypothesis-generating evidence of temporal molecular precedence but is not confirmatory.
MEL-4	Discordant / Non-informative	No convincing molecular lead time can be demonstrated because molecular progression is absent, discordant with radiological progression, or the longitudinal trajectory is insufficient for meaningful evaluation.	Persistent molecular decline until PD, isolated analytical fluctuations, absence of molecular progression, persistent PD without evaluable response phase, or inadequate follow-up.	Radiological PD occurs without preceding molecular progression, or no evaluable lead-time interval exists.	No meaningful evidence supporting molecular lead time.	No informative pre-radiological molecular lead-time signal was established for that episode.

Supplementary Table S8. Patient-level Molecular Lead-Time Analysis and Molecular Evidence Level (MEL) Adjudications. Patient-level adjudication of all evaluable progression episodes using the MEL framework. The table identifies the RECIST trajectory, the specific molecular-progression criterion and patient-specific evidence supporting each mPD assignment, the corresponding molecular and radiological progression dates, and the resulting molecular lead time (MLT). MEL-4 includes episodes with concurrent molecular and radiological progression, no qualifying pre-radiological molecular-progression event, or molecular–radiological discordance.

MEL	PID	RECIST Trend	Qualifying mPD criterion and evidence	Interpretation	mPD date	First rPD date	MLT (days)
**MEL-1**	**14152**	PD, SD, PD	≥50% rise above running nadir: 0.36% → 3.5%; qualifying VAF > assay LoD	Clear post-response molecular relapse preceded RECIST-defined PD with a sustained and biologically coherent increase in ctDNA.	09-03-18	06-04-18	**28**
**MEL-1**	**14313**	PD, SD, PD	LoD-qualified molecular re-emergence: prior molecular-negative / near-negative state → 0.42% > LoD	Molecular progression clearly developed before RECIST PD with a reproducible upward trajectory.	16-04-18	01-06-18	**46**
**MEL-1**	**14355**	PD, SD, PR, PD	≥50% rise above running nadir: 0.32% → 0.81%; qualifying VAF > LoD	One of the clearest examples of sustained molecular relapse substantially preceding radiological progression.	11-05-18	02-08-18	**83**
**MEL-1**	**15928**	PD, SD, PR, PD	Confirmed negative-to-positive conversion: two consecutive 0% measurements → 0.036% > LoD	Molecular re-emergence during radiological response preceded RECIST-defined PD, representing a clear molecular relapse before imaging progression.	13-12-18	16-02-19	**65**
**MEL-1**	**17463**	PD, SD, PD	≥50% rise above running nadir: 0.028% → 0.27%; qualifying VAF > LoD	Molecular deterioration became evident during SD before subsequent RECIST progression.	03-11-18	07-12-18	**34**
**MEL-2**	**14173**	PD, SD, PD	≥50% rise above running nadir: 0.05% → 0.40%; qualifying VAF > LoD; subsequent oscillatory course	Low-level oscillatory increases eventually preceded PD despite a less distinct molecular trajectory.	04-05-18	29-11-18	**209**
**MEL-2**	**14630**	PD, SD, PD	Negative-to-positive conversion: consecutive negative measurements → 0.06% positive signal	Molecular re-emergence preceded PD but longitudinal confirmation was limited by sparse sampling.	06-04-18	26-05-18	**50**
**MEL-3**	**15329**	PD, SD, PR, PD, SD	LoD-associated molecular re-emergence: two 0% measurements → 0.047% > LoD	Molecular behavior was compatible with progression but remained analytically borderline because values fluctuated around the assay LoD.	02-08-18	24-10-18	**83**
**MEL-3**	**16972**	PD, SD, PD	≥50% rise above running nadir: 0.17% → 0.33% (+94.1%); 0.33% > LoD 0.02%	Persistent molecular positivity preceded PD but the trajectory remained less definitive than MEL-1 or MEL-2.	17-11-18	11-01-19	**55**
**MEL-4**	**14134**	PD, SD, PD	≥50% rise above running nadir, concurrent with rPD: 0.07% → 0.15%; >LoD at rPD	No measurable molecular lead time because molecular and radiological progression occurred at the same assessment.	31-03-18	31-03-18	**0**

MEL	PID	RECIST Trend	Qualifying mPD criterion and evidence	Interpretation	mPD date	First rPD date	MLT (days)
**MEL-4**	**18102**	PD, SD, PR, PD	No qualifying pre-rPD mPD: low-level concurrent re-emergence at rPD (0.012%), below LoD 0.02%	Minimal molecular re-emergence occurred concurrently with RECIST-defined PD, providing no measurable molecular lead time.	24-01-19	24-01-19	**0**
**MEL-4**	**14307**	PD, SD, PR, PD	≥50% rise above running nadir, concurrent with rPD: 1.3% → 6.8%; >LoD at rPD	Excellent molecular response was observed during PR; however, molecular relapse occurred concurrently with radiological progression, yielding no measurable lead time.	28-02-19	28-02-19	**0**
**MEL-4**	**15730**	PD, SD, PD	No qualifying pre-rPD mPD: LoD-associated fluctuations did not satisfy the qualifying progression rule	Radiological progression occurred without a reproducible molecular progression event.	n.a.	n.a.	n.a.
**MEL-4**	**17798**	PD, SD, PD	No qualifying mPD: sustained molecular decline despite subsequent rPD	ctDNA continued to decline despite subsequent RECIST-defined progression, demonstrating molecular–radiological discordance.	n.a.	n.a.	n.a.

mPD criteria: (i) ≥50% increase above the running VAF nadir with the qualifying VAF exceeding the assay-specific LoD; or (ii) confirmed molecular-negative to molecular-positive conversion, as defined in Methods. For episodes without a qualifying pre-rPD mPD, the reason for nonqualification is shown. LoD, limit of detection; mPD, molecular progression; rPD, radiological progression; MLT, molecular lead time; n.a., not applicable.

Supplementary Table S9. Exploratory landmark analyses of early molecular response, molecular clearance, and survival outcomes. Association of early molecular response (≥50% VAF reduction) and molecular clearance at the 60- and 90-day landmarks with progression-free survival (PFS) and overall survival (OS). Hazard ratios (HRs) were estimated relative to molecular nonresponders. Molecular-clearance analyses are presented descriptively because sparse events precluded reliable Cox model estimation. CI, confidence interval; HR, hazard ratio; NE, not estimable.

Molecular endpoint	Landmark	Outcome	Evaluable patients	Endpoint positive	Endpoint negative	Events, positive / negative	HR	95% CI	Cox *p*	Log-rank *p*
≥50% VAF reduction	Day 60	PFS	41	25	16	9 / 9	0.53	0.20–1.38	0.191	0.184
		OS	42	25	17	16 / 14	0.69	0.34–1.42	0.315	0.312
	Day 90	PFS	35	21	14	6 / 7	0.42	0.13–1.34	0.144	0.132
		OS	38	22	16	14 / 13	0.54	0.25–1.16	0.116	0.111
Molecular clearance	Day 60	PFS	41	5	36	1 / 17	NE	—	—	0.045
		OS	42	5	37	4 / 26	NE	—	—	0.833
	Day 90	PFS	35	6	29	1 / 12	NE	—	—	0.061
		OS	38	6	32	4 / 23	NE	—	—	0.363

Table Footnote: All p-values are nominal and unadjusted for multiplicity. Molecular-clearance comparisons are descriptive owing to sparse events; in particular, the day-60 PFS comparison included five clearance-positive patients and one subsequent PFS event and is not interpreted as confirmatory.

Supplementary Table S10. Primer and probe sequences for the 36 unique personalized ddPCR assay designs used in the clinical cohort. Sequences are reported 5′→3′. Fluorophores used were 6-carboxyfluorescein (FAM) for the mutant probes and 2′-chloro-7′-phenyl-1,4-dichloro-6-carboxyfluorescein (VIC) for the wild type probe. Quenchers were either Black Hole Quencher-1 (BHQ-1) or Minor Groove Binder Non-Fluorescent Quencher (MGB-NFQ).

Gene	Variant	Forward primer	Reverse primer	Mutant probe	WT probe
AKT1	E17K	GCCATCATTCTTGAGGAGGAAGTAG	GAGTGTGCGTGGCTCTCA	AGGTCTTGATGTACTTCCCTA	CAGGTCTTGATGTACTCCCCTA
APC	L1489fs*18	TGCAGTTCAGAGGGTCCAGG	GATGGATTTTCTTGTTCATC	GATACTTTATACATTTTG	GATACTTTATTACATTTTG
BRCA2	R2784W	ATCAATATATTTATTAATTT	TGGATTCTTTCCTGACCCTA	TAACAGTACTTGGCCTGCT	TAACAGTACTCGGCCTGCT
CDKN2A	Y129*	CGGGCATGGTTACTGCCTCT	GCTCCTCAGCCAGGTCCACG	CGCGCGCAGTTACCGTGCG	CGCGCGCAGGTACCGTGCG
EGFR	D855N	TGAAAACACCGCAGCATGTC	CGCACCCAGCAGTTTGG	AGATCACAAATTTTG	AAGATCACAGATTTTG
EGFR	E746-A50del	TGGATCCCAGAAGGTGAGAA	TCCTCGATGTGAGTTTCTGC	GTCGCTATCAAGACATCTCCGAA	GTCGCTATCAAGGAATTAAGAGAAGCAACATCTCCGAAAGCCAA
EGFR	L858R	GCAGCATGTCAAGATCACAGATT	CCTCCTTCTGCATGGTATTCTTTCT	AGTTTGGCCCGCCCAA	AGTTTGGCCAGCCCAA
EGFR	T790M	CGCCTGCTGGGCATCT	TCTTTGTGTTCCCGGACATAGTC	ATGAGCTGCATGATGAG	ATGAGCTGCGTGATGAG
FGFR3	S249C	GGGAGGGGGTGGCCCCTGAG	GGGCTGCCGGCCAACCAGAC	CAGAGCGCTGCCCGCACCGG	CAGAGCGCTCCCCGCACCGG
GNAS	R201C	CTTTGGTGAGATCCATTGACCTCAA	CACCTGGAACTTGGTCTCAAAGATT	CAGGACACAGCAGCGA	CAGGACACGGCAGCGA
GNAS	R201H	CTTTGGTGAGATCCATTGACCTCAA	CACCTGGAACTTGGTCTCAAAGATT	CGCTGCCATGTCCT	TTCGCTGCCGTGTCCT
GNAS	R844H	AGATCCATTGACCTCAATTT	ACCAAGTTCCAGGTGGACAA	CGCTGCCATGTCCTGACT	CGCTGCCGTGTCCTGACT
IDH1	R132H	CCAACATGACTTACTTGATCCCCATA	CTTGTGAGTGGATGGGTAAAACCTA	ATCATAGGTCATCATGC	CATCATAGGTCGTCATGC
KRAS	G12A	GCTGTATCGTCAAGGCACTCTT	AGGCCTGCTGAAAATGACTGAATAT	TTGGAGCTGCTGGCGTA	TTGGAGCTGGTGGCGTA
KRAS	G12V	GATTCTGAATTAGCTGTATC	TTCAGTCATTTTCAGCAGGC	CCTACGCCAACAGCTCCAAC	CCTACGCCACCAGCTCCAAC
KRAS	G13D	GAATTAGCTGTATCGTCAAGGCACT	AGGCCTGCTGAAAATGACTGAATAT	CTTGCCTACGTCACCAG	CTTGCCTACGCCACCAG
NOTCH1	G1704R	GATCTTGTAGGGGATGTTGA	CTCTGGAAGCACTGCGAGGA	CGAGCGCTCTCAGGAATGC	CGAGCGCTCCCAGGAATGC
NRAS	G12D	GCTGTATCGTCAAGGCACTCTT	AGGCCTGCTGAAAATGACTGAATAT	TTGGAGCTGATGGCGTA	TTGGAGCTGGTGGCGTA
NRAS	Q61L	CATGTACTGGTCCCTCATTGCA	GATGGAGAAACCTGTCTCTTGGAT	ACTCCTCTAGACCTGC	TGTACTCCTCTTGACCTGC
PIK3CA	E542K	GGGAAAATGACAAAGAACAGCTCAA	GCACTTACCTGTGACTCCATAGAAA	CCTCTCTCTAAAATCA	CCTCTCTCTGAAATCA

Gene	Variant	Forward primer	Reverse primer	Mutant probe	WT probe
PIK3CA	E545K	TCAAAGCAATTTCTACACGAGATCCT	GCACTTACCTGTGACTCCATAGAAA	CTCTGAAATCACTAAGCAG	CTCTCTGAAATCACTGAGCAG
PIK3CA	H1047L	GCAAGAGGCTTTGGAGTATTTCATG	GCTGTTTAATTGTGTGGAAGATCCAA	CACCATGAAGTGCATC	CCACCATGATGTGCATC
PIK3CA	H1047R	GCAAGAGGCTTTGGAGTATTTCATG	GCTGTTTAATTGTGTGGAAGATCCAA	CACCATGACGTGCATC	CCACCATGATGTGCATC
PIK3CA	K111E	AATTGAACCAGTAGGCAACCGT	ATGGTTATTTGCATTTTAGAATAGGATATTGTATCA	CGATTGAGGATCTCTTCTT	TCGATTGAGGATCTTTTCTT
TP53	G266V	CTCTCCCAGGACAGGCACAA	AGGAAAAGAGAAGCAAGAGG	CTGTTCCGTACCAGTAGATT	CTGTTCCGTCCCAGTAGATT
TP53	I254N	CAGGGTGGCAAGTGGCTCCT	GCCGCCCATGCAGGAACTGT	GTGTGATGTTGGTGAGGAT	GTGTGATGATGGTGAGGAT
TP53	K132E	CCACAGCTGCACAGGGCAGG	AAGAGGAAGGAGACAGAGTT	CAAAACATCTCGTTGAGGG	CAAAACATCTTGTTGAGGG
TP53	K132R	CCCACAGCTGCACAGGGCAG	GAAGAGGAAGGAGACAGAGT	AAAACATCCTGTTGAGGG	AAAACATCTTGTTGAGGG
TP53	R156P	CCGTCATGTGCTGTGACTGC	AACCCACAGCTGCACAGGGC	GCGCGGACGGGGGTGCCGG	GCGCGGACGCGGGTGCCGG
TP53	R175H	CTGCTCACCATCGCTATCTGA	ACAGCACATGACGGAGGTT	TGAGGCACTGCCC	TGAGGCGCTGCCC
TP53	R248Q	CCAGTGTGATGATGGTGAGGAT	AACTACATGTGTAACAGTTCCTGCAT	CATGAACCAGAGGCC	CATGAACCGGAGGCC
TP53	R248W	GAGTCTTCCAGTGTGATGATGGT	AACTACATGTGTAACAGTTCCTGCAT	TGGGCCTCCAGTTCA	TGGGCCTCCGGTTCA
TP53	R249S	GAGTCTTCCAGTGTGATGATGGT	AACTACATGTGTAACAGTTCCTGCAT	AACCGGAGTCCCATCC	CCGGAGGCCCATCC
TP53	R273H	CTGTGCGCCGGTCTCT	GGTAATCTACTGGGACGGAACAG	TTGAGGTGCATGTTTGT	TGAGGTGCGTGTTTGT
TP53	R282W	CTTTCTTGCGGAGATTCTCTTCCT	GCTTTGAGGTGCGTGTTTGTG	TGCGCCAGTCTCT	TGCGCCGGTCTCT
TP53	R342P	TCCCAGCCTGGGCATCCTTG	ACGGATCTGCAGCAACAGAG	TTCAGCTCTGGGAACATCT	TTCAGCTCTCGGAACATCT
